# Expanding the Ambient-Pressure Phase Space of CaFe_2_O_4_-Type Sodium Postspinel Host–Guest Compounds

**DOI:** 10.1021/acsorginorgau.1c00019

**Published:** 2021-09-01

**Authors:** Justin C. Hancock, Kent J. Griffith, Yunyeong Choi, Christopher J. Bartel, Saul H. Lapidus, John T. Vaughey, Gerbrand Ceder, Kenneth R. Poeppelmeier

**Affiliations:** † Department of Chemistry, Northwestern University, Evanston, Illinois 60208, United States; ‡ Joint Center for Energy Storage Research, Argonne National Laboratory, Argonne, Illinois 60439, United States; § Department of Materials Science and Engineering, University of California, Berkeley, California 94720, United States; ∥ X-ray Science Division, Argonne National Laboratory, Argonne, Illinois 60439, United States; ⊥ Chemical Sciences and Engineering Division, Argonne National Laboratory, Lemont, Illinois 60439, United States; # Materials Sciences Division, Lawrence Berkeley National Laboratory, Berkeley, California 94720, United States

**Keywords:** postspinel, calcium ferrite, tunnel structure, energy storage, complex oxides

## Abstract

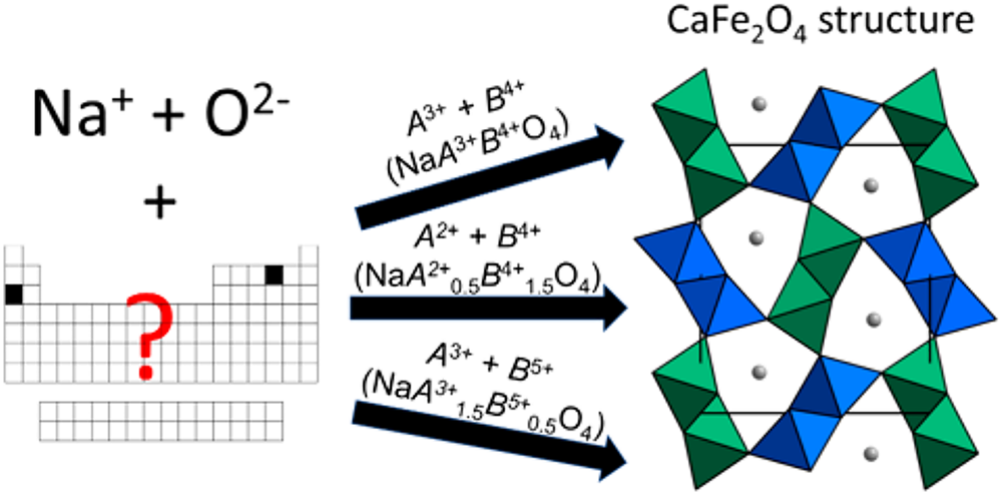

CaFe_2_O_4_-type sodium postspinels (Na-CFs), with Na^+^ occupying tunnel sites, are of interest as prospective battery electrodes. While many compounds of this structure type require high-pressure synthesis, several compounds are known to form at ambient pressure. Here we report a large expansion of the known Na-CF phase space at ambient pressure, having successfully synthesized NaCrTiO_4_, NaRhTiO_4_, NaCrSnO_4_, NaInSnO_4_, NaMg_0.5_Ti_1.5_O_4_, NaFe_0.5_Ti_1.5_O_4_, NaMg_0.5_Sn_1.5_O_4_, NaMn_0.5_Sn_1.5_O_4_, NaFe_0.5_Sn_1.5_O_4_, NaCo_0.5_Sn_1.5_O_4_, NaNi_0.5_Sn_1.5_O_4_, NaCu_0.5_Sn_1.5_O_4_, NaZn_0.5_Sn_1.5_O_4_, NaCd_0.5_Sn_1.5_O_4_, NaSc_1.5_Sb_0.5_O_4_, Na_1.16_In_1.18_Sb_0.66_O_4_, and several solid solutions. In contrast to earlier reports, even cations that are strongly Jahn–Teller active (e.g., Mn^3+^ and Cu^2+^) can form Na-CFs at ambient pressure when combined with Sn^4+^ rather than with the smaller Ti^4+^. Order and disorder are probed at the average and local length-scales with synchrotron powder X-ray diffraction and solid-state NMR spectroscopy. Strong ordering of framework cations between the two framework sites is not observed, except in the case of Na_1.16_In_1.18_Sb_0.66_O_4_. This compound is the first example of an Na-CF that contains Na^+^ in both the tunnel and framework sites, reminiscent of Li-rich spinels. Trends in the thermodynamic stability of the new compounds are explained on the basis of crystal-chemistry and density functional theory (DFT). Further DFT calculations examine the relative stability of the CF versus spinel structures at various degrees of sodium extraction in the context of electrochemical battery reactions.

## Introduction

**Figure 1:**
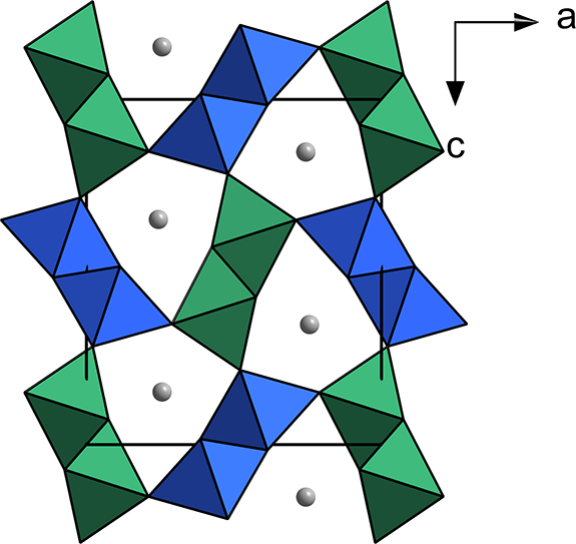
CaFe_2_O_4_ structure. Gray spheres represent Na^+^. Green and blue octahedra represent the two symmetrically independent framework sites comprising the two “double rutile” chains.

The CaFe_2_O_4_ (calcium ferrite or CF) structure, also occasionally referred to as the CaV_2_O_4_ structure because it was identified first for this compound,^1,2^ has been of increasing interest in recent years. Previously, the structure type was primarily of geological and crystal-chemical relevance.^3−8^ Many spinel compounds transform to this structure type under high pressure (hence the CF structure is termed “post-spinel”), and the structure is thought be a host for various cations in the Earth’s mantle.^3−10^ However, many property- and application-oriented studies have been recently published, indicating renewed interest in this class of materials.^11−28^ Materials that crystallize in the CF structure (see Figure 1) have been reported with interesting magnetic and electronic properties resulting from its pseudo-1D chain structure and geometric frustration.^11−15^ The large tunnels in CF compounds have led to their investigation as host structures for phosphors^16−18^ and battery cathode materials.^19−28^ CF host structures have been shown to function well as both Na and Li cathode materials,^19,20,23,24^ and Mg ions are also predicted to be mobile in the tunnels.^26,27^

The calcium ferrite (CF) structure is composed of a framework of octahedrally coordinated cations, arranged into “double-rutile” chains that run parallel to the *b* axis. Within the chains, the octahedra are edge-sharing, and the chains connect to adjacent chains via corner-sharing. There are two crystallographically distinct metal sites in the framework, with each double chain containing only one unique site, paired by symmetry, as illustrated in Figure 1. The chains surround one-dimensional channels of 8-coordinate cations. For the CF materials synthesizable at ambient pressure, the tunnel sites have been reported to be occupied by Na^+^, Ca^2+^, Sr^2+^, and Ba^2+^.^29^ CF compounds with Na^+^ occupying the tunnel sites (compounds hereafter referred to as Na-CFs) are the most likely to be relevant to the energy storage materials community because of the relatively high mobility of Na^+^. Divalent Ca^2+^ is expected to show low mobility,^28^ and Sr^2+^ and Ba^2+^ primarily form CF structures when the framework cations are redox inactive trivalent rare-earth metals.^29^ However, Na-CF materials with redox-active transition metal cations have been synthesized at ambient pressure, although the library of such compounds, detailed in the following paragraph, is limited.

The only comprehensive study of the crystal chemistry of Na-CF phases was performed by Reid, Wadsley, and Sienko, who studied compounds of the formula Na*A*^3+^*B*^4+^O_4_ using high-temperature solid-state synthesis.^8^ They synthesized and evaluated NaScTiO_4_, NaFeTiO_4_, NaFeSnO_4_, NaScZrO_4_, NaScHfO_4_, and NaAlGeO_4_, the last of which required high pressure. Other compositions, such as NaMnTiO_4_, were attempted but resulted in no CF phases. Based on these results, Reid et al. concluded that “spherical” (Jahn–Teller inactive) ions favor formation of a CF phase. Several new compounds of this type have since been reported,^25,30−32^ although many require high pressure for synthesis, such as NaAlSiO_4_, NaV_2_O_4_, NaCr_2_O_4_, NaMn_2_O_4_, and NaRh_2_O_4_.^5,6,11,12,33,34^ Na-CF compounds with other stoichiometries have also been shown to exist, including Na*A*^2+^_0.5_*B*^4+^_1.5_O_4_ (*A*^2+^ = Co^2+^, Ni^2+^ and *B*^4+^ = Ti^4+^) and Na*A*^3+^_1.5_*B*^5+^_0.5_O_4_ (*A*^3+^ = Fe^3+^ and *B*^5+^ = Sb^5+^), all of which have been synthesized at ambient pressure.^35−37^ The few Na-CFs deviating significantly from these stoichiometries were synthesized via hydrothermal synthesis, including Na_0.55_Fe_0.28_Ti_1.72_O_4_ and Na_3_Mn_4_Te_2_O_12_ (Na[Mn_1.33_Te_0.67_]O_4_), the latter having a superstructure owing to crystallographic ordering of Te^6+^ and Mn^2+/3+^.^38,39^ Notably, some of the CF phases synthesized at ambient pressure contain ions that are weakly Jahn–Teller active, thus the spherical ion preference appears not to be a strict criterion. This would suggest many more as yet undiscovered Na-CF compounds may be stable even at ambient pressure.

In this paper, we report a large expansion of the ambient-pressure phase space of Na-CF materials and critically examine the crystal chemical relationships. Notably, we successfully synthesized several new Na-CFs with redox-active transitions metals, which are prospective Na/Li/Mg battery electrode materials, and Na_1.16_In_1.18_Sb_0.66_O_4_, the first Na-CF that contains sodium on both the tunnel and framework sites.

## Experimental Section

### Synthesis

All compounds were synthesized via high-temperature solid-state reactions. The synthesis temperature depended on the composition of the CF phase. Unless stated otherwise, starting materials were NaHCO_3_ and binary metal oxides. In a typical synthesis, appropriate amounts of these reactants were mixed with a mortar and pestle then pressed into a pellet at 400 MPa. The pellet was placed in either a platinum crucible (air syntheses) or platinum boat (inert atmosphere syntheses), heated at the specified temperature for 2 days, ground into a powder, and repeated as necessary. Some compositions (those containing Cr^3+^, Mn^2+^, and Fe^2+^) required an inert atmosphere for synthesis. These samples were heated in a tube furnace under flowing argon, with a titanium rod placed upstream to remove any residual O_2_. In some cases, excess NaHCO_3_ was added in the subsequent annealing steps to account for sodium loss from volatilization. All reactions carried out in air (except NaMnSnO_4_, for which we were reproducing a reported synthesis) were quenched on the benchtop to reflect the thermodynamics of the synthesis temperature and minimize cooling rate effects. All reactions carried out in a tube furnace were cooled by shutting off the power once the dwell step completed. Detailed synthetic information for each new compound is discussed in more detail in the Results and Discussion section, and reaction conditions for combinations of cations that did not produce a CF phase are included in Table S1.

### X-ray Diffraction

Phase purity was assessed by laboratory powder X-ray diffraction (PXRD) using both Rigaku Ultima IV and Rigaku SmartLab X-ray diffractometers. These data were collected over a 2θ range of 10–60° under ambient conditions. Synchrotron X-ray radiation was used for Rietveld refinements, except in the case of NaFe_0.5_Ti_1.5_O_4_, NaMn_0.5_Sn_1.5_O_4_, NaFe_0.5_Sn_1.5_O_4_, and NaCd_0.5_Sn_1.5_O_4_. These data were collected at 11-BM at the Advanced Photon Source at Argonne National Laboratory using a wavelength of 0.45788 Å (∼27 keV). This wavelength was chosen to minimize absorption of X-rays by In and Sn. Samples were packed into Kapton capillaries.

Rietveld refinement was performed using the General Structure Analysis System II (GSAS II) package.^40^ Ionic scattering factors were used in all cases. All data were refined against an orthorhombic unit cell (space group *Pnma*). In all cases, the unit cell parameters, atomic coordinates, and *U*_iso_ values were refined. In the case of NaCd_0.5_Sn_1.5_O_4_ only, *U*_iso_ for the oxygen atoms was not refined and set to a reasonable value of 0.01. An 8- or 10-term Chebyshev polynomial was used to fit the backgrounds, with an added background peak to account for scattering from the Kapton capillary. To decrease the degrees of freedom, global compositions were fixed, and atomic positions and isotropic thermal parameters were constrained to be equal for all atoms sharing the same crystallographic sites. In cases where cation ordering of the framework sites seemed probable (see the Results and Discussion section), occupancies were refined, but in most cases occupation of the framework sites was assumed to be statistically distributed to simplify the model and avoid overfitting.

### Solid-State NMR Spectroscopy

^23^Na solid-state NMR spectra were recorded at 9.4 T (ν_L_(^23^Na) = 105.7 MHz) with a Bruker Avance III spectrometer and a Bruker HX probe. Samples were packed into a 4.0 mm diameter (80 μL volume) zirconia rotor with a Kel-F cap and measured at ambient temperature under 12.5 kHz magic-angle spinning (MAS), corresponding to approximately 30 °C, unless otherwise noted. One-dimensional spectra were collected with a one-pulse (Bloch decay) sequence and a 2.0 μs (π/4)_liquid_ pulse. NaCl (aqueous, 1.0 M) was used to optimize the solution π/2 pulse and as the ^23^Na reference at 0 ppm. In all cases, the recycle delay was set to at least 5 × *T*_1_, where *T*_1_ is measured with a saturation-recovery pulse sequence. Typical *T*_1_ relaxation times were 0.5–8 s. The multiple-quantum magic-angle spinning (MQMAS) spectrum of Na_1.16_In_1.18_Sb_0.66_O_4_ was recorded with a z-filtered pulse sequence with excitation and conversion pulses of 9.0 and 3.0 μs followed by a 28 μs selective pulse.^41^ Acquisition in the indirect dimension comprised 192 *t*_1_ increments of 25 μs. For each *t*_1_-slice, 396 scans were averaged and the recycle delay was 0.6 s, resulting in an experimental time of 12.7 h.

### DFT Calculations

Calculations is this work are based on density functional theory (DFT) as implemented in the Vienna Ab Initio Simulation Package (VASP)^42^ using the projector augmented-wave method^43,44^ and the generalized gradient approximation as formulated by Perdew, Burke, and Ernzerhof.^45^ For all calculations, the energy cutoff was set to 520 eV, and at least 1000 k-points were used per reciprocal atom. For geometry optimizations, energies were converged to 10^–5^ eV for electronic steps and 10^–4^ eV for ionic steps. The Hubbard U correction was used for the transition metal atoms to account for the self-interaction error of semilocal density functionals.^46^ U parameters were chosen to be consistent with the Materials Project database,^47^ as reported by Jain et al. (Co, 3.32 eV; Cr, 3.7 eV; Fe, 5.3 eV; Mn, 3.9 eV; Ni, 6.2 eV).^48^

Supercells of the spinel (*Fd*3̅*m*) and CF postspinels (*Pnma*) with 32 oxygen ions (Na_*x*_(*A*,*B*)_16_O_32_) were used for all calculations, and the ionic positions, cell shape, and volume were allowed to optimize during relaxations. The cell size was chosen to provide enough ions for sampling various occupations for Na and Na vacancies on the alkali site as well as *A* and *B* on the octahedral sies. To generate ordered structures, the lowest and second lowest electrostatic energy configurations (as calculated with the Ewald method) were sampled to order the *A* and *B* cations on the octahedral sites and determine which Na sites to remove. In most compositions, two different configurations were used but in slow converging composition only the lowest configuration was used for DFT calculation. In total, 264 different structures were calculated at varying levels of sodiation (*x*) to determine the thermodynamic stability of each phase in the spinel and postspinel structures. Thermodynamic stability was determined using the convex hull method, where the formation energies of all competing phases in each Na-*A*-*B*-O chemical space were taken from the Materials Project database. The pymatgen library was used to set up and analyze the calculations in this work.^49^

## Results and Discussion

The Na-CF chemical space was systematically explored using numerous cation combinations and stoichiometries, and this approach and the results are summarized schematically in Figure 2. Sixteen new Na-CF end-member compounds were successfully synthesized, and NaMnSnO_4_, reported recently by Chiring et al.,^25^ was reexamined. Rietveld refinement data for these compounds are shown in Table 1, and an example Rietveld refinement is shown in Figure 3. Additional Rietveld refinements are shown in Figures S1–S16.

**Figure 2:**
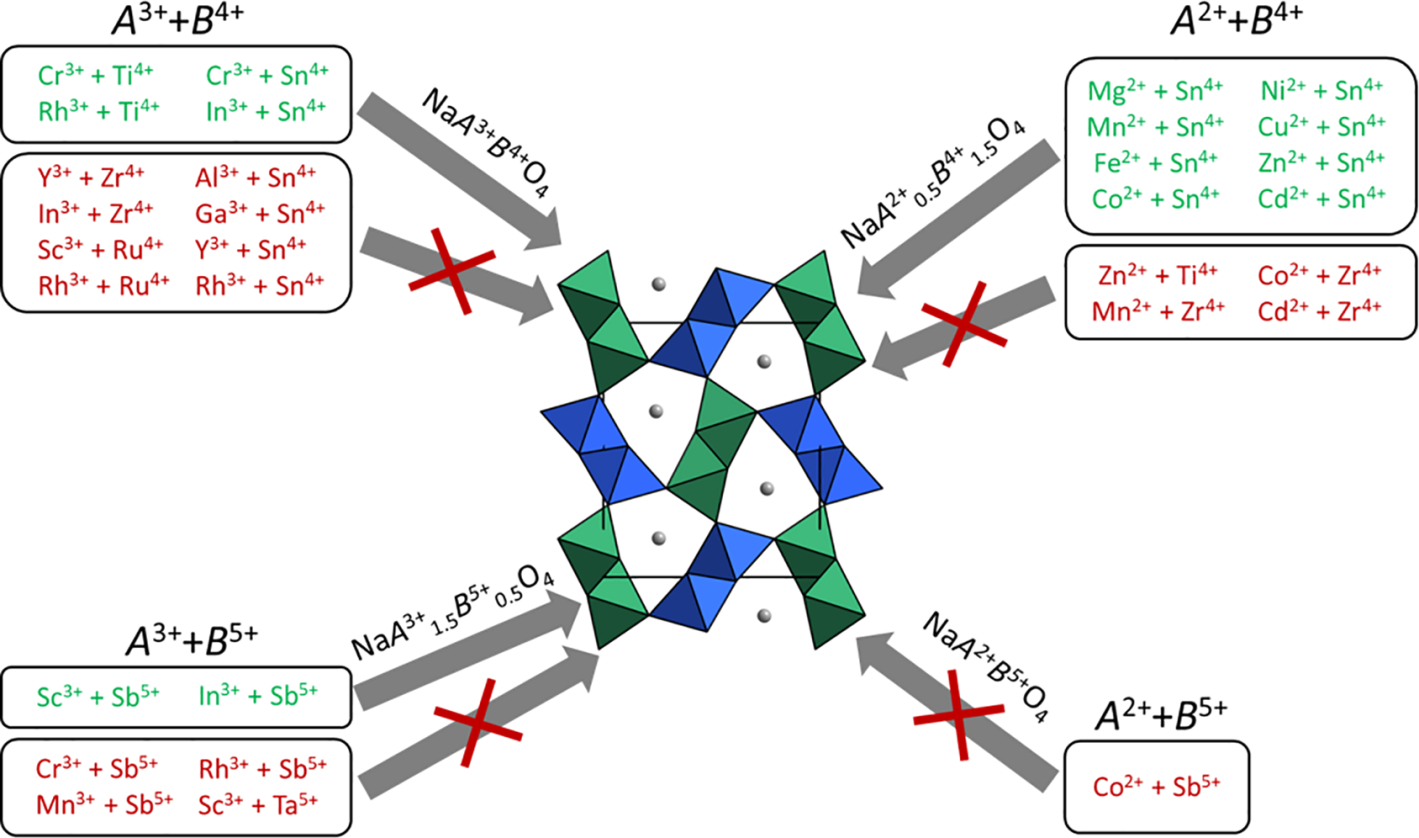
Schematic summarizing the synthetic approach and results presented in this paper. The ion combinations in green were successfully synthesized in the postspinel structure, whereas the ion combinations in red formed alternative phases.

**Table 1:** Rietveld Refinement Data for New Na-CF Compounds

source	synchrotron					
chemical formula	Na_0.99_Cr_0.99_Ti_1.01_O_4_	Na_0.96_Rh_0.96_Ti_1.04_O_4_	NaCrSnO_4_	NaMnSnO_4_	Na_0.96_In_0.96_Sn_1.04_O_4_	NaMg_0.5_Ti_1.5_O_4_
formula weight	186.61	234.67	257.67	260.61	319.73	170.99
temperature (K)	298					
wavelength (Å)	0.457880					
crystal system	orthorhombic					
space group (no.)	*Pnma* (62)					
*a* (Å)	9.101854(15)	9.16953(5)	9.26744(3)	9.42779(10)	9.53203(3)	9.17179(2)
*b* (Å)	2.933813(4)	2.947138(15)	3.048247(9)	3.02517(3)	3.172342(8)	2.968472(6)
*c* (Å)	10.668108(17)	10.79754(5)	10.93396(4)	11.11389(11)	11.29355(3)	10.76171(2)
α = β = γ (deg)	90					
*V* (Å^3^)	284.872(1)	291.791(3)	308.878(2)	316.976(7)	341.504(2)	290.001(1)
Z	4					
profile range	3 ≤ 2θ ≤ 37.9963					
GOF	1.93	1.04	2.03	2.38	2.46	1.40
*R*_p_ (%)	6.82	10.60	7.71	9.92	6.55	6.27
*R*_wp_ (%)	9.70	12.88	9.72	12.44	9.10	7.37

source	synchrotron					
chemical formula	NaMg_0.5_Sn_1.5_O_4_	NaCo_0.5_Sn_1.5_O_4_	NaNi_0.5_Sn_1.5_O_4_	NaCu_0.5_Sn_1.5_O_4_	NaZn_0.5_Sn_1.5_O_4_	NaSc_1.5_Sb_0.5_O_4_
formula weight	277.17	294.49	294.37	296.79	297.71	215.3
temperature (K)	298					
wavelength (Å)	0.457880					
crystal system	orthorhombic					
space group (no.)	*Pnma* (62)					
*a* (Å)	9.41987(3)	9.41576(3)	9.39739(3)	9.47695(3)	9.43720(5)	9.44848(6)
*b* (Å)	3.106399(8)	3.115976(8)	3.099946(7)	3.101415(7)	3.113964(15)	3.133693(18)
*c* (Å)	11.11941(3)	11.10660(3)	11.10428(3)	11.08428(3)	11.13601(6)	11.13570(7)
α = β = γ (deg)	90					
*V* (Å^3^)	325.375(2)	325.859(2)	323.483(2)	325.789(2)	327.255(4)	329.713(4)
*Z*	4					
profile range	3 ≤ 2θ ≤ 37.9963					
GOF	1.34	1.85	1.54	2.01	1.66	2.08
*R*_p_ (%)	7.48	6.31	6.96	6.48	8.79	9.83
*R*_wp_ (%)	8.96	8.33	9.10	8.26	12.45	12.40

source	synchrotron	Cu Kα			
chemical formula	Na_1.16_In_1.18_Sb_0.66_O_4_	NaFe_0.5_Ti_1.5_O_4_	NaMn_0.5_Sn_1.5_O_4_	NaFe_0.5_Sn_1.5_O_4_	NaCd_0.5_Sn_1.5_O_4_
formula weight	306.51	186.76	292.49	292.94	321.23
temperature (K)	298	298			
wavelength (Å)	0.457880	1.5406			
crystal system	orthorhombic	orthorhombic			
space group (no.)	*Pnma* (62)	*Pnma* (62)			
*a* (Å)	9.53383(2)	9.2175(3)	9.48122(13)	9.40435(15)	9.5604(2)
*b* (Å)	3.168104(6)	2.96553(9)	3.13771(4)	3.11440(5)	3.17096(7)
*c* (Å)	11.28573(2)	10.7674(4)	11.19800(15)	11.10497(18)	11.2812(3)
α = β = γ (deg)	90	90			
*V* (Å^3^)	340.876(1)	294.33(2)	333.133(9)	325.252(11)	341.995(17)
*Z*	4	4			
profile range	3 ≤ 2θ ≤ 37.9963	10 ≤ 2θ ≤ 130			10 ≤ 2θ ≤ 120
GOF	2.16	1.81	2.26	2.42	2.55
*R*_p_ (%)	8.65	1.84	3.34	2.83	3.99
*R*_wp_ (%)	11.61	2.55	4.45	3.96	5.36

**Figure 3:**
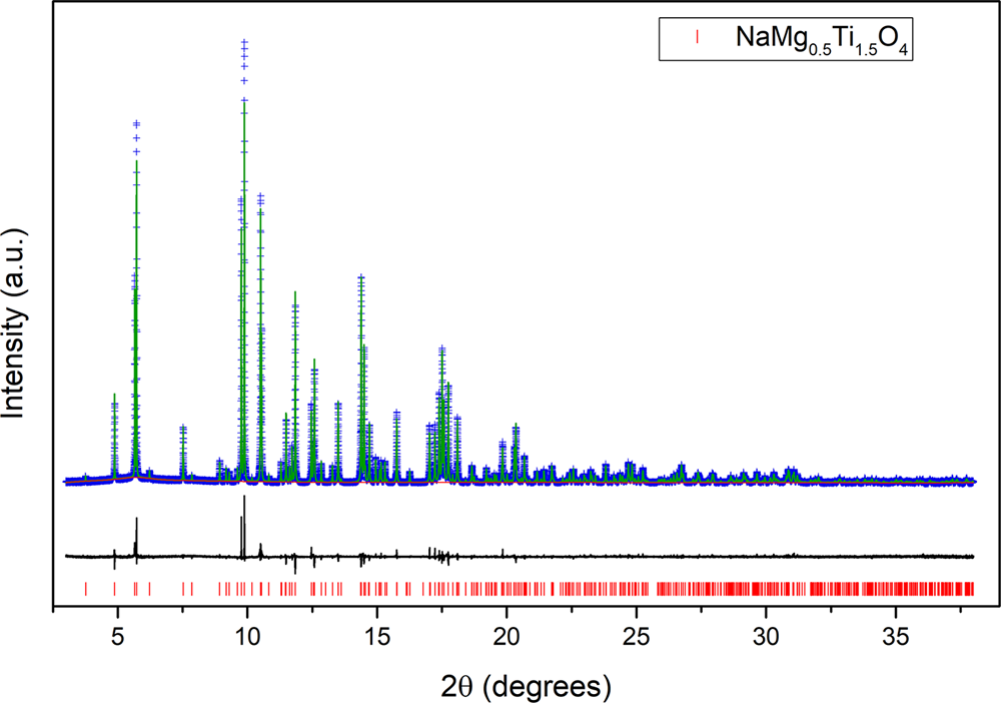
Rietveld refinement using synchrotron data for NaMg_0.5_Ti_1.5_O_4_. Blue crosses are the observed intensities, the green curve is the fitted pattern, the black curve is the difference pattern, and the red tick marks indicate the location of the CF-NaMg_0.5_Ti_1.5_O_4_ peaks.

### Synthetic Studies

#### New Postspinels in the Na^+^-*A*^3+^-*B*^4+^-O^2–^ System (Idealized Formula: Na*A*^3+^*B*^4+^O_4_)

**Figure 4:**
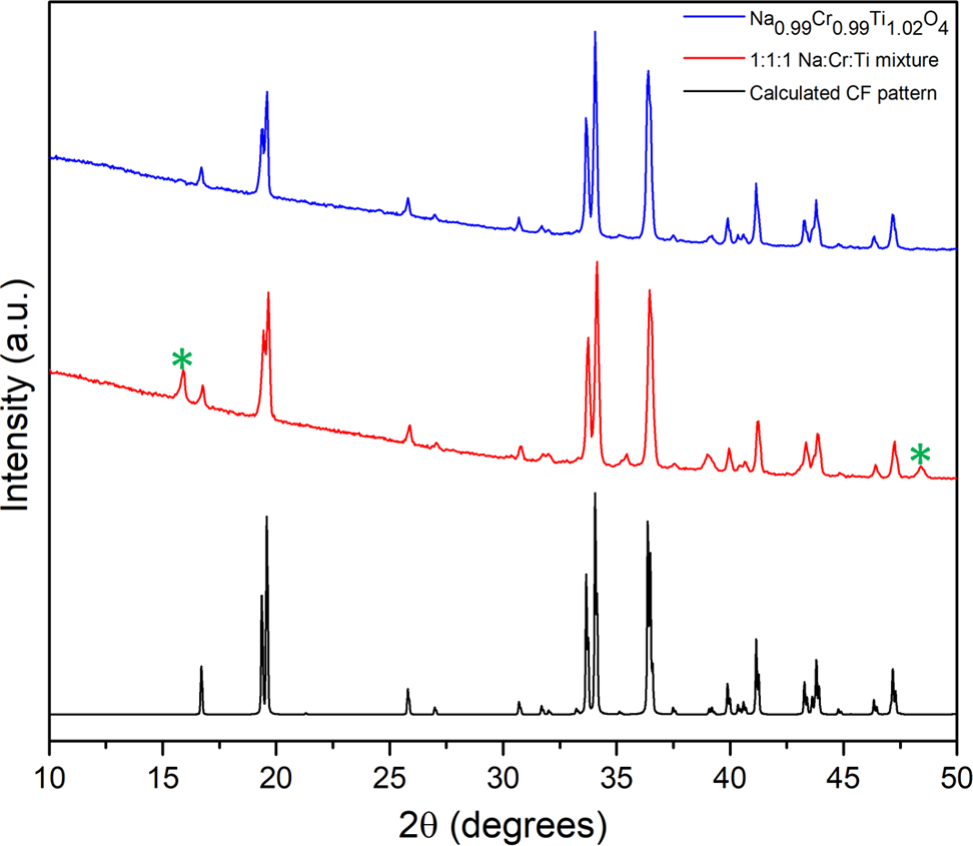
Powder XRD patterns for the 1:1:1 and 0.99:0.99:1.01 Na/Cr/Ti samples compared to a calculated pattern for the CF phase. Asterisks highlight peaks from the secondary phase Na_0.6_Cr_0.6_Ti_0.4_O_2_ in the 1:1:1 Na/Cr/Ti sample.

The new CF phase NaCrTiO_4_ was successfully synthesized at ambient pressure. NaCrTiO_4_ must be synthesized in an inert atmosphere (Ar in this case) to avoid the oxidation of Cr^3+^ to Cr^6+^ and formation of Na_2_CrO_4_. Initially, reactions performed in the temperature range of 875–900 °C were used to minimize sodium volatility. For a 1:1:1 Na/Cr/Ti ratio, after 48 h a mixture of three quaternary phases formed, which included a CF phase, layered Na_1–*x*_Cr_1–*x*_Ti_*x*_O_2_, probably close to the Na_0.6_Cr_0.6_Ti_0.4_O_2_ composition reported by Avdeev et al.,^50^ and Na_*x*_Cr_*x*_Ti_2–*x*_O_4_, with a nonstoichiometric sodium iron titanate (NSIT) structure. The NSIT phase probably has a composition close to Na_0.9_Cr_0.9_Ti_1.1_O_4_, which is the reported upper limit of *x* found in the Na_*x*_Fe_*x*_Ti_2–*x*_O_4_ system.^51^ (Note that, confusingly, NSIT phases are sometimes referred to as CaV_2_O_4_-type structures in databases, but we emphasize that the NSIT structure is distinct from the CF structure.) While the quaternary phases formed relatively quickly, they reacted very slowly with each other, and the system was slow to reach thermal equilibrium once these phases formed. Heating the mixture for an additional 48 h at 900 °C increased the percentage of CF-NaCrTiO_4_, but the other two phases were still present. Higher temperatures sped up the reaction, and the loss of Na from volatility appears to be minimal at 950 °C. At this temperature, the NSIT phase was no longer observed. Instead, the stoichiometric mixture (1:1:1 Na/Cr/Ti) resulted in Na_0.6_Cr_0.6_Ti_0.4_O_2_ and the CF compound (see Figure 4), the relative ratios of which changed little upon further heating. This suggests that the CF compound is nonstoichiometric and is deficient in both sodium and chromium relative to the ideal composition NaCrTiO_4_. Addition of ∼2% excess TiO_2_ to this mixture and further annealing at 900 °C led to a nearly single-phase product, suggesting an actual composition of Na_0.99_Cr_0.99_Ti_1.01_O_4_ for the CF phase. A separate mixture synthesized at 950 °C with 5% TiO_2_ excess still contained some Na_0.6_Cr_0.6_Ti_0.4_O_2_, suggesting the composition of the CF phase may have some degree of temperature dependency. Because the competing phases have such similar stoichiometries, the ratio of cations must be carefully controlled to avoid significant fractions of the non-CF phases.

The synthesis of NaCrSnO_4_ requires higher temperatures than that of NaCrTiO_4_. The formation of the CF phase was slow even at 950 °C, and NaCrO_2_ and SnO_2_ were the main phases after 48 h. Heating at 1000 °C produced CF-NaCrSnO_4_ as the main phase, but NaCrO_2_, potentially Sn-substituted, also formed, and SnO_2_ was still present at shorter reaction times. Reheating these mixtures again at 1000 °C resulted in elimination of the SnO_2_ phase, but with similar proportions of NaCrO_2_ and CF-NaCrSnO_4_. This suggests a loss of Sn. In some runs, metallic Sn was observed. Cleaning the alumina tube alleviated this problem somewhat, suggesting reductive species build up over time in the tube, potentially from the Ti rod, used as a getter, and its interaction with volatile phases. Nevertheless, phase-pure NaCrSnO_4_ was never obtained by this synthetic method alone, even when using excess SnO_2_, but the pure CF phase could likely be formed in a closed system such as a sealed metal tube. However, purification was possible. NaCrO_2_ was removed by treating the mixture with molten KNO_3_, which selectively oxidized the NaCrO_2_. The soluble Cr(VI) products were then dissolved in water, and the remaining solid was filtered, leaving behind only CF-NaCrSnO_4_.

Recently, CF-NaMnSnO_4_ was reported to be synthesizable under ambient pressure in air.^25^ The authors reported that, when using a stoichiometric starting composition, phase purity was achieved only by slowly cooling at 0.5 °C/min after heating at 1200 °C for a day. Similarly, we observed secondary phases upon quenching stoichiometric mixtures but obtained a nearly phase-pure CF compound upon quenching a sample with a Na/Mn/Sn ratio of 0.96:0.96:1.04. The lattice parameters for the quenched and slowly cooled samples are significantly different, suggesting the difference in composition is real. These results, like those for the Na–Cr–Ti–O system, suggest a temperature dependence of the composition of the CF phase. Interestingly, both the quenched and slowly cooled samples had broad peaks with poorer Rietveld fits compared to the other compositions, probably caused by a high degree of strain. Presumably, this strain results from substituting a strongly JT-active cation (Mn^3+^) that prefers highly distorted octahedral environments into sites that usually contain more spherically symmetric cations.

CF-NaRhTiO_4_ was synthesized in air from NaRhO_2_ (prepared by heating NaHCO_3_ and metallic Rh powder at 900 °C for about 1 day) and TiO_2_. A layered phase similar to the one formed in the Na–Cr–Ti-O system, Na_*x*_Rh_1–*x*_Ti_*x*_O_2_ forms quickly, with subsequent slow formation of the CF phase at 900 °C. Increasing the temperature to 950 °C increases the rate of formation, and the CF phase is the major phase after 48 h. However, heating at 1050 °C destabilizes the CF phase. At this temperature, no CF phase is observed, and the layered phase is the primary phase along with another unknown phase present in small amounts. As in the case of Na_1–*x*_Cr_1–*x*_Ti_1+*x*_O_4_, phase purity was not achieved with the ideal stoichiometry. In fact, the product of the mixture with a 1:1:1 ratio of cations had a nearly identical PXRD pattern to that of the 1:1:1 ratio in the Na–Cr–Ti–O system. Phase purity was achieved by using an excess of titanium relative to the ideal composition, and the phase-pure sample had a nominal composition of Na_0.96_Rh_0.96_Ti_1.04_O_4_. CF-NaRhSnO_4_ did not form under similar conditions up to a temperature of 1100 °C.

CF-NaInSnO_4_ appears to be more refractory than the CF phases containing Ti^4+^ and could be synthesized in the 950–1200 °C temperature range. The sample with the highest phase purity was synthesized by using a Na/In/Sn ratio of 0.98:0.96:1.04 and air-quenching from 1200 °C, the highest temperature studied, after ∼20 h. Thus, the actual composition of the CF phase is likely close to Na_0.96_In_0.96_Sn_1.04_O_4_, and presumably, the excess Na was lost through volatilization. Thus, all the new CF compounds of the type Na*A*^3+^*B*^4+^O_4_ deviate slightly from the ideal stoichiometry, with the possible exception of NaCrSnO_4_, whose precise composition was not determined owing to the difficulty in obtaining a phase-pure sample. CF-NaInZrO_4_ did not form under similar conditions.

#### New Postspinels in the Na^+^-*A*^2+^-*B*^4+^-O^2–^ System (Idealized Formula: Na*A*^2+^_0.5_*B*^4+^_1.5_O_4_)

The compounds NaMg_0.5_Ti_1.5_O_4_ and NaFe_0.5_Ti_1.5_O_4_ can be synthesized with high phase purity. A stoichiometric mixture of NaHCO_3_, MgO, and TiO_2_ heated at 925 °C for 48 h resulted in primarily Na_0.9_Mg_0.45_Ti_1.55_O_4_ (NSIT-type structure). Phase-pure NaMg_0.5_Ti_1.5_O_4_ was not obtained until the mixture was further heated at 950 °C for ∼96 h with an intermediate grinding step. The CF phase decomposes after 3 h at 1050 °C into NSIT-Na_0.9_Mg_0.45_Ti_1.55_O_4_, layered Na_0.68_Mg_0.34_Ti_0.66_O_2_, and MgO. The presence of MgO suggests Na volatilization at this temperature. Interestingly, NaMg_0.5_Ti_1.5_O_4_ was not reported in a previous study of the Na–Mg–Ti–O system that found six phases, possibly because of the fast formation of the NSIT and layered phase and limited temperature range in which the CF phase can be formed.^52^ NaFe_0.5_Ti_1.5_O_4_ was readily synthesized in one step at 925 °C with a 5% excess of sodium from Na_8_Ti_5_O_14_, FeTiO_3_, and TiO_2_ under flowing argon. The relatively fast kinetics of this synthesis could be explained by the use of different reactants. This phase seems somewhat air-sensitive even at room temperature. The sample used for synchrotron diffraction had highly asymmetric peaks skewed toward higher angles after being stored for ∼3 months, suggesting topotactic oxidation (Fe^2+^ to Fe^3+^). With this observation, a new sample was synthesized and laboratory X-ray data were collected for Rietveld refinement. It should be pointed out that a CF phase containing Fe^2+^ has been reported to form via hydrothermal synthesis, but the phase has a different composition (Na_0.55_Fe_0.28_Ti_1.72_O_4_) and is unusually Na-deficient.^38^ In addition, a “CF-like” secondary phase was mentioned in a study of the NSIT phases Na_*x*_Fe^2+^_*x*/2_Ti^4+^_2–*x*/2_O_4_ for higher values of *x* but was not discussed further.^53^ No other divalent cations (Mn^2+^, Cu^2+^, or Zn^2+^) could be fully substituted into Na*A*^2+^_0.5_Ti^4+^_1.5_O_4_ under similar synthetic conditions.

No CF phases with the composition Na*A*^2+^_0.5_Sn_1.5_O_4_ have been previously reported. However, more compositions of this type could be synthesized than in the Na*A*^2+^_0.5_Ti_1.5_O_4_ system; Mg^2+^, Mn^2+^, Fe^2+^, Co^2+^, Ni^2+^, Cu^2+^, Zn^2+^, and Cd^2+^ can all form CF compounds when combined with Sn^4+^. These compounds are more refractory than the corresponding compounds with Ti^4+^, so a wider range of temperatures (950–1200 °C) was used in the synthesis of the Na*A*^2+^_0.5_Sn_1.5_O_4_ compounds. The compounds Na*A*^2+^_0.5_Sn_1.5_O_4_ (*A*^2+^ = Mg^2+^, Co^2+^, Ni^2+^) were first heated at 1200 °C for 1 day with a 5% excess of sodium, then reground with an additional 5% excess of sodium (as NaHCO_3_) and reheated at 1000 °C for 2 days. The excess Na decreased the amount of SnO_2_ observed as a minor secondary phase. The compounds Na*A*^2+^_0.5_Sn_1.5_O_4_ (*A*^2+^ = Mn^2+^, Fe^2+^, Zn^2+^, Cd^2+^) were synthesized at 1000 °C for 48 h, and NaCu_0.5_Sn_1.5_O_4_ was synthesized at 950 °C for 96 h. NaMn_0.5_Sn_1.5_O_4_ and NaFe_0.5_Sn_1.5_O_4_ were synthesized under flowing argon, and Fe^2+^ was introduced as Fe(C_2_O_4_)·2H_2_O. In most cases, near phase purity was achieved, although it was difficult to eliminate SnO_2_ as a secondary phase (e.g., ∼1% by weight in the case of NaCo_0.5_Sn_1.5_O_4_). NaZn_0.5_Sn_1.5_O_4_ always formed along with secondary phases, though in the best sample, the ratio of the most intense CF peak of the PXRD pattern to the most intense secondary phase peak was slightly greater than 13:1.

While a very small degree of deviation from ideal stoichiometry is possible, no systematic trends were observed during synthesis of the Na*A*^2+^_0.5_*B*^4+^_1.5_O_4_ compounds that would suggest sodium vacancies, in contrast to the Na*A*^3+^*B*^4+^O_4_ compounds.

#### New Postspinels in the Na^+^-*A*^3+^-*B*^5+^-O^2–^ System (Idealized Formula: Na*A*^3+^_1.5_*B*^5+^_0.5_O_4_)

CF phases were also found in the Na–In–Sb–O and Na–Sc–Sb–O systems. Attempts to synthesize materials of the ideal compositions NaIn_1.5_Sb_0.5_O_4_ and NaSc_1.5_Sb_0.5_O_4_ always resulted in a CF phase and either In_2_O_3_ or Sc_2_O_3_. Decreasing the ratio of In_2_O_3_ to the other reactants (NaHCO_3_ and Sb_2_O_3_) improved the phase purity, suggesting the composition of the CF phase is better represented by the formula Na_1+*x*_In_1.5–2*x*_Sb_0.5+*x*_O_4_. When *x* = 0.158, a single-phase CF material was obtained after heating at 1200 °C for 1 day, followed by quenching. Thus, In^3+^ appears to be cosubstituted by Sb^5+^ and Na^+^. We were not able to make the pure CF phase in the Na–Sc–Sb–O system using the same strategy. The best sample was synthesized at 1100 °C for 96 h with an intermediate grinding and with the ideal ratio of cations (i.e., 1:1.5:0.5 ratio of Na to Sc to Sb), but this sample contained Sc_2_O_3_ and another unknown phase as secondary phases. NaCr_1.5_Sb_0.5_O_4_ did not form at 900 or 950 °C under flowing argon and from a mixture of NaHCO_3_, NaSbO_3_, and Cr_2_O_3_.

### Solid Solutions

**Figure 5:**
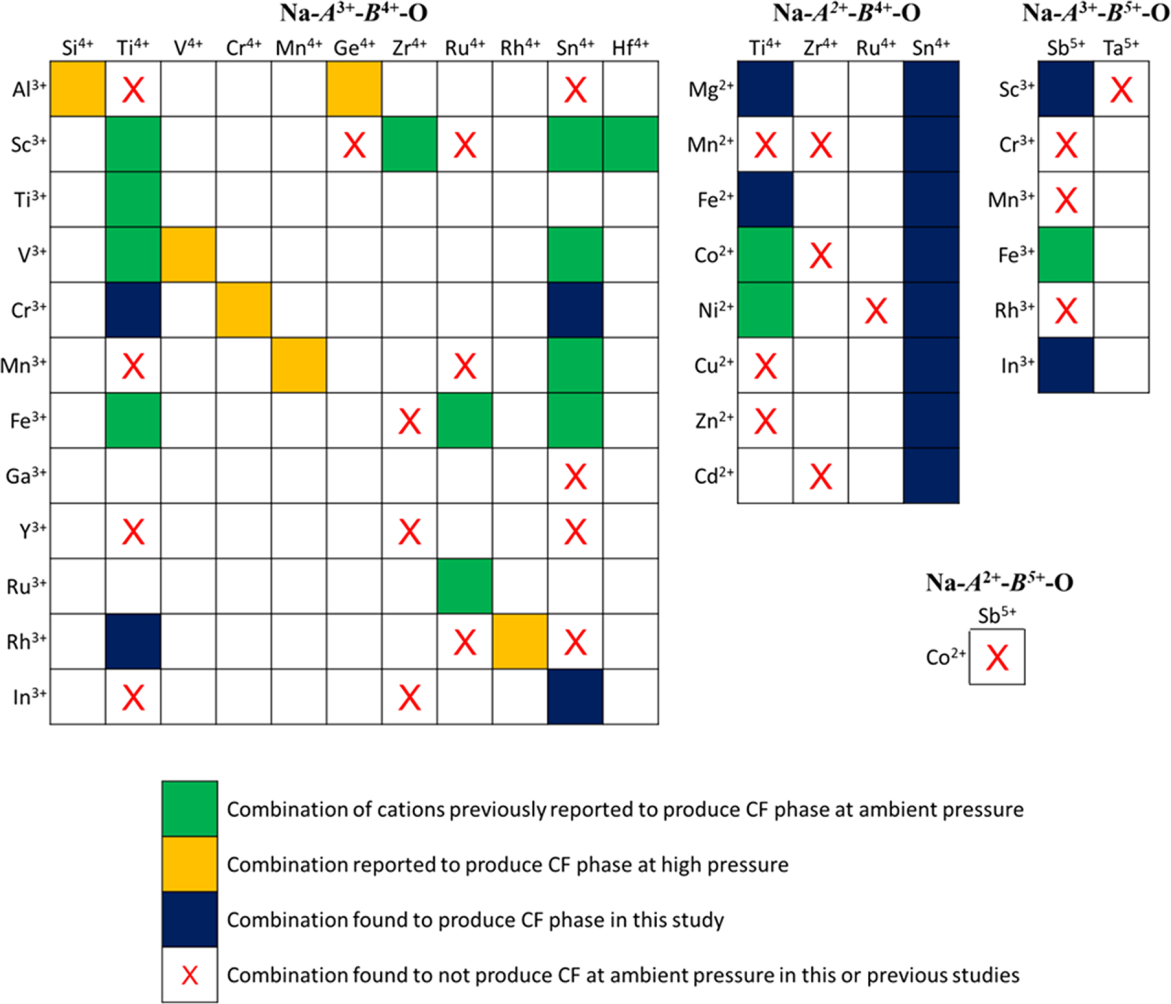
A combinatorial representation of the Na-CF phase space. The phase-space shown covers all Na-CF compounds and combinations known to have been attempted in previous studies or this work. Conceivable but unexplored combinations (e.g., Sc^3+^ and Mo^4+^) are not depicted.

Owing to similarities in synthesis conditions, ability to move from ideal cation locations, and nonstoichiometry, solid solutions between the systems Na*A*^3+^*B*^4+^O_4_ and Na*A*^2+^_0.5_*B*^4+^_1.5_O_4_ should exist. Several compositions were tried, including NaCo_1/3_Fe_1/3_Ti_4/3_O_4_, NaNi_1/3_Fe_1/3_Ti_4/3_O_4_, and NaNi_1/3_Sc_1/3_Ti_4/3_O_4_, which produced CF phases with no apparent secondary phases. NaCo_1/3_Fe_1/3_Ti_4/3_O_4_ and NaNi_1/3_Fe_1/3_Ti_4/3_O_4_ were synthesized at 900 °C for 96 h with intermediate grindings. NaNi_1/3_Sc_1/3_Ti_4/3_O_4_ also formed at these temperatures and was stable at 1050 °C, in contrast to the other Na-CFs containing Ti. The flexibility of these phases with respect to compositional tuning further emphasizes their potential usefulness for applications such as intercalation cathodes for energy storage. The results summarizing the now-expanded Na-CF phase space (excluding solid solutions) are shown in Figure 5.

### Crystal Chemistry

#### Cation Distribution

The Na-CF material in the Na–In–Sb–O system was obtained phase pure with the nominal stoichiometry Na_1.16_In_1.18_Sb_0.66_O_4_ (Na_1+*x*_In_1.5–2*x*_Sb_0.5+*x*_O_4_, *x* = 0.16). This would imply a cosubstitution of Na^+^ and Sb^5+^ for In^3+^, which would be the first reported instance of Na^+^ occupying the framework sites of a CF compound. Rietveld refinements for this material provide further evidence of this new cation distribution. The CF structure contains two independent octahedral cation sites, which are referred to here to as the *M1* and *M2* sites. Assuming an actual composition of NaIn_1.5_Sb_0.5_O_4_ resulted in unreasonable (negative) thermal parameters (*U*_iso_) for the *M2* site, even when the occupancy of Na^+^ in the tunnel sites was refined. Refining the composition Na[Na_0.16_In_1.18_Sb_0.66_]O_4_ with complete disorder of the framework sites also resulted in a negative *U*_iso_ for the *M2* site. Refining the fractional occupancies of the framework sites and constraining the composition alleviated this problem. Because three atoms distributed between two sites is an underdetermined system using only X-ray data and because In^3+^ and Sb^5+^ have nearly equal scattering factors, Sb^5+^ was treated as In^3+^ for this refinement. (Note that for the final refinement and in the CIF, Sb^5+^ was reintroduced such that the In/Sb ratio was the same for both sites.) By this refinement, nearly all (∼90%) of the octahedral Na^+^ occupies the *M1* site rather than the *M2* site, with a final distribution of Na[(In/Sb)_0.856(2)_Na_0.144(2)_]^*M1*^[(In/Sb)_0.984(2)_Na_0.016(2)_]^*M2*^O_4_. A model placing Na^+^ on the *M2* site only and not allowing for occupancy refinement results in a significantly worse fit. The results of these refinements are summarized in Table 2. This Na-CF with Na^+^ occupying the framework sites is analogous to lithium-rich spinels such as Li_4_Mn_5_O_12_ (Li[Li_1/3_Mn_5/3_]O_4_) and the commercial lithium-ion battery material Li_4_Ti_5_O_12_ (Li[Li_1/3_Ti_5/3_]O_4_).^54,55^

**Table 2:** Refinement Statistics for Different Structural Models of Na_1.16_In_1.18_Sb_0.66_O_4_

starting cation distribution model	*M1* and *M2* site occupancies refined?	*R*_wp_ (%)	comment
Na[In_0.75_Sb_0.25_]^*M1*^[In_0.75_Sb_0.25_]^*M2*^O_4_	no	11.90	negative *U*_iso_ for *M2*
Na[In_0.59_Sb_0.33_Na_0.08_]^*M1*^[In_0.59_Sb_0.33_Na_0.08_]^*M2*^O_4_	no	11.97	negative *U*_iso_ for *M2*
Na[In_0.92_Na_0.08_]^*M1*^[In_0.92_Na_0.08_]^*M2*^O_4_	yes	11.61	reasonable *U*_iso_s, 90% Na on *M1* site
Na[In]^*M1*^[In_0.84_Na_0.16_]^*M2*^O_4_	no	13.30	negative *U*_iso_ for *M2*

**Figure 6:**
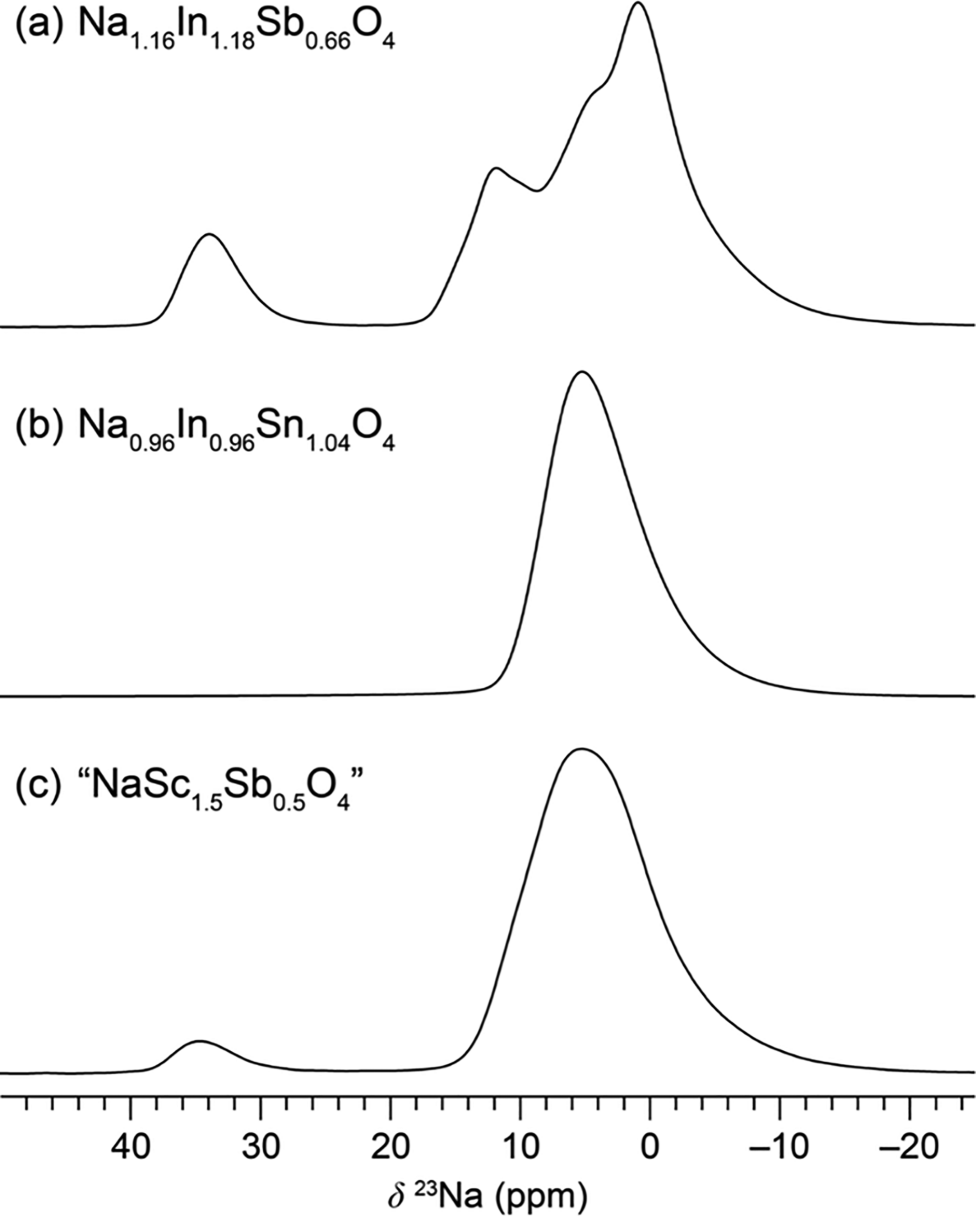
^23^Na solid-state NMR spectra at 12.5 kHz MAS and 9.4 T: (a) Na_1.16_In_1.18_Sb_0.66_O_4_, (b) Na_0.96_In_0.96_Sn_1.04_O_4_, and (c) “NaSc_1.5_Sb_0.5_O_4_” central transition resonances.

The sodium environments in Na_1.16_In_1.18_Sb_0.66_O_4_ were further investigated with ^23^Na solid-state magic-angle spinning (MAS) NMR spectroscopy. The ^23^Na NMR spectrum of Na_1.16_In_1.18_Sb_0.66_O_4_, shown in Figure 6a, contains a series of overlapping resonances at lower frequencies (−10 to +20 ppm), with an additional resonance at +34 ppm. The overlapping low-frequency resonances, which resolve into at least four signals with multiple-quantum magic-angle spinning (MQMAS) (Figure S17), likely correspond to Na in the tunnel sites. Although there is crystallographically only one tunnel site, the local environment is more complex given the multiple next-nearest neighbor possibilities. Multiple peaks for the tunnel sites are also resolved in some other CF phases (see Figure S18). The higher shift of the signal at 34 ppm is consistent with octahedral Na^+^, and the integration of this peak (11%) is reasonably consistent with the fraction of Na expected to occupy the octahedral sites. Another possibility that could explain the ^23^Na NMR spectrum is that the stoichiometry is ideal, NaIn_1.5_Sb_0.5_O_4_, but with Na^+^-In^3+^ antisite defects analogous to inversion in spinels. This is sensible crystal-chemically, as In^3+^ does occupy 8-coordinate sites in some phases.^56^ If this did occur in NaIn_1.5_Sb_0.5_O_4_, it would also be expected to occur in NaInSnO_4_. However, the ^23^Na NMR spectrum for Na_0.96_In_0.96_Sn_1.04_O_4_, shown in Figure 6b, contains only the low-frequency signal distribution. Thus, we concluded antisite defects were unlikely to be the cause of the signal at 34 ppm for Na_1.16_In_1.18_Sb_0.66_O_4_. A third possibility is that the high frequency resonance is from an unidentified, and possibly amorphous, secondary phase. In the nominally stoichiometric NaIn_1.5_Sb_0.5_O_4_ sample, PXRD showed only CF and In_2_O_3_ as crystalline phases, and the ^23^Na NMR spectrum matched that of Na_1.16_In_1.18_Sb_0.66_O_4_. Since this sample is Na-poor, it is unlikely that the resonance at 34 ppm could come from a Na-containing secondary phase. The ^23^Na NMR spectra for “NaSc_1.5_Sb_0.5_O_4_” and “NaCd_0.5_Sn_1.5_O_4_” also show weak resonances at 35 ppm (Figure 6 and Figure S18), and these phases also probably contain framework sodium. While the CF phase in the Na–Sc–Sb–O system is likely also not quite stoichiometric NaSc_1.5_Sb_0.5_O_4_, a Rietveld refinement assuming this stoichiometry gave reasonable *U*_iso_ values when framework occupancies were refined. However, the ^23^Na NMR spectrum for the “NaSc_1.5_Sb_0.5_O_4_” sample (Figure 6c) contained a small peak at 35 ppm that integrates to ∼4.5% of the total Na. There was a secondary phase that we were unable to identify so we cannot conclusively say that this peak corresponds to framework-site Na in the CF phase, but it is likely given the parallels with the Na–In–Sb–O system. If “NaSc_1.5_Sb_0.5_O_4_” does deviate from the ideal stoichiometry, the difference is not as large as in Na_1.16_In_1.18_Sb_0.66_O_4_, and corefinements with neutron data may be necessary to determine the cation distribution accurately.

While the CF structure contains two crystallographically independent octahedral cation sites, each double-chain contains only one of these sites (see Figure 1). Reid et al. found no evidence of cation ordering in the Na-CFs they synthesized.^8^ A single-crystal study of NaFeRuO_4_ also found no site preference for the framework cations Fe^3+^ and Ru^4+^.^31^ Only two studies of hydrothermally synthesized Na-CFs have found any statistically significant site preference.^38,57^ In these cases, the synthesis temperature is considerably lower than typically used for solid-state synthesis. As noted by Reid et al., the intensities of most of the X-ray reflections are insensitive to ordering. However, the (101) reflection is strongly affected by site preference and has negligible intensity in the absence of ordering and/or when the framework cations have similar scattering factors. Thus, in this study, occupancies of the framework cations were only refined when the (101) peak was apparent in the PXRD pattern to avoid overfitting.

None of the compounds reported here have strong (101) reflections present, but a weak reflection (note the small peak at ∼3.5° in Figure 3) indicating some degree of site preference is apparent for NaMg_0.5_Ti_1.5_O_4_, NaCu_0.5_Sn_1.5_O_4_, Na_1.16_In_1.18_Sb_0.66_O_4_, and NaSc_1.5_Sb_0.5_O_4_. That the CF phase obtained in the Na–In–Sb–O system has a (101) reflection is further evidence that it is not the initially expected NaIn_1.5_Sb_0.5_O_4_ (In^3+^ and Sb^5+^ are essentially indistinguishable by X-rays). One might expect that cation site preference is more likely when cation radii and charge density differences are larger or when JT-active cations like Mn^3+^ and Cu^2+^ are present, since these cations are expected to prefer different coordination environments than Ti^4+^ or Sn^4+^. Of the Na-CF compounds reported here, only NaMnSnO_4_ and NaCu_0.5_Sn_1.5_O_4_ contain cations with strong Jahn–Teller distortions. The (101) reflection is essentially nonexistent in the case of NaMnSnO_4_, even for the slowly cooled sample, thus there is no evidence of site preference. For NaCu_0.5_Sn_1.5_O_4_, refinement of the occupancies of the framework sites indicated that ∼63% of the Cu^2+^ occupies the *M1* site, with Cu occupancies of 0.317(2) and 0.183(2) for the *M1* and *M2* sites, respectively. Likewise, ∼63% of the Mg^2+^ cations occupy the *M1* site in NaMg_0.5_Ti_1.5_O_4_, with Mg occupancies of 0.316(1) and 0.184(1) on the *M1* and *M2* sites, respectively. The strongest cation preference was observed for Na_1.158_In_1.184_Sb_0.658_O_4_. In this instance, ∼88% of the octahedral Na^+^ cations sit on the *M1* site according to the Rietveld refinement. In^3+^ and Sb^5+^ could preferentially occupy either of the sites too, but this cannot be determined by X-ray diffraction. NaSc_1.5_Sb_0.5_O_4_ also has a weak (101) reflection, and cation site preference is likely in this compound. However, because we do not know with certainty the amount of Na^+^ occupying the framework sites in this compound, it is difficult to identify the mechanism of this partitioning. Given these results, ordering of the framework cations in Na-CFs seems to be driven by differences in cationic radii and charge density rather than JT activity. It would be expected, then, that NaCd_0.5_Sn_1.5_O_4_ would show cation site preference. However, X-rays cannot distinguish between Cd^2+^ and Sn^4+^, thus the cation distribution could not be fully examined in the present work; a detailed NMR crystallography study of this question is ongoing. Neutron diffraction studies would also be a valuable complement to the work presented here, as X-ray data alone is insufficient for occupancy studies when the framework atoms differ little in electron count (e.g., NaCrTiO_4_ and NaCd_0.5_Sn_1.5_O_4_). Furthermore, each of the samples with cation site preference were quenched from the synthesis temperature. It is possible that annealing at lower temperature would increase the degree of site preference, as lower synthesis temperatures (hydrothermal synthesis) resulted in site preference in the Na–Fe–Ti–O CFs,^38,57^ which was not observed for the NaFe_0.5_Ti_1.5_O_4_ compound synthesized by a solid-state reaction and presented in this paper.

### Phase Space and Compositional Trends

CF-NaCr_2_O_4_, NaMn_2_O_4_, and NaRh_2_O_4_ have all been synthesized in the postspinel structure but required the use of high pressure.^12,32,33^ The requirement of high pressure is likely due to a combination of factors. The compounds are mixed-valent, and the oxidation states of Cr and Rh therein are unusual; Rh^4+^ compounds often require high-pressure and highly oxidizing environments, while Cr^4+^ compounds also often require high pressure to avoid disproportionation into Cr^3+^ and Cr^6+^. Mn^4+^ can be formed at ambient pressures, but Mn^3+^ is strongly Jahn–Teller active. Thus, CF-NaMn_2_O_4_ would not be expected to be stable by the criteria suggested by Reid et al., which potentially explains the necessity of high pressure. Ionic radius also appears to play a role, and it should also be noted that no Na-CF synthesized under ambient pressure contains framework cations smaller than 0.60 Å (the size of Sb^5+^)^58^ as major components. Rh^4+^ (0.60 Å) is similar in size to Sb^5+^, but both Cr^4+^ (0.55 Å) and Mn^4+^ (0.53 Å) are smaller. However, Cr^3+^ (0.615 Å) and Rh^3+^ (0.665 Å) are JT-inactive (spherical), stable, and have ionic radii consistent with cations known to form CF compounds at ambient pressure. Thus, we reasoned that replacement of Cr^4+^ and Rh^4+^ with a more stable and larger tetravalent cation like Ti^4+^ (0.605 Å) or Sn^4+^ (0.69 Å) should increase the chance of synthesizing a CF compound at ambient pressure. This strategy was successful in the synthesis of NaV^3+^(V_0.25_Ti_0.75_)^4+^O_4_ and NaVSnO_4_.^31^ Indeed, it was found that Cr^3+^ can form a Na-CF phase when paired with either Ti^4+^ or Sn^4+^. Rh^3+^ formed a Na-CF phase when combined with Ti^4+^, though we were unable to make CF-NaRhSnO_4_. Reid et al. found that CF-NaMnTiO_4_ did not form at ambient pressure, and this result was among the evidence that the CF structure tends to form with spherical ions. Instead, a mixture corresponding to the stoichiometry NaMnTiO_4_ forms Na_4_Mn_4_Ti_5_O_18_ at ambient pressure.^59^ This structure contains two types of tunnels: a smaller tunnel with a shape reminiscent of those found in the CF structure, and a larger S-shaped tunnel. Half of the Mn^3+^ cations are 5-coordinate in this structure. Likewise, “NaMn_2_O_4_” forms the isostructural Na_4_Mn_9_O_18_ at ambient pressure.^60^ Interestingly, replacing Ti^4+^ with Sn^4+^ results in CF-NaMnSnO_4_.

No Na-CF compound containing In^3+^ has previously been reported. Reid et al. attempted to synthesize NaInTiO_4_ but obtained a mixture of In_2_O_3_ and Na_2_Ti_3_O_7_.^8^ Synthesis of NaInZrO_4_ was attempted in this work because NaScZrO_4_ was reported and the size mismatch would be decreased, but that did not form either. However, NaInSnO_4_ was successfully synthesized. It should be noted that the ionic radius of In^3+^ (0.800 Å) is larger than Sc^3+^ (0.745 Å), which means In^3+^ is the largest trivalent cation known to form a Na-CF compound at ambient pressure. Y^3+^ (0.900 Å) is apparently too large. The compound NaYTiO_4_ has a layered perovskite structure instead of the CF structure.^61^ In the yttrium system, replacing Ti^4+^ with Sn^4+^ or Zr^4+^ still did not produce a CF phase.

Only two compounds of the type Na*A*^2+^_0.5_*B*^4+^_1.5_O_4_ have been reported previously: NaCo_0.5_Ti_1.5_O_4_ and NaNi_0.5_Ti_1.5_O_4_.^35,36^ Given the trends established by Reid et al., other combinations of metal cations should be possible. It was found that Co^2+^ and Ni^2+^ could be replaced by Mg^2+^ (JT-inactive) or Fe^2+^ (weakly JT-active). No CF phases formed when the compositions Na*A*^2+^_0.5_Ti_1.5_O_4_ (*A*^2+^ = Mn^2+^, Cu^2+^, and Zn^2+^) were targeted. That CF-NaCu_0.5_Ti_1.5_O_4_ could not be synthesized at ambient pressure is consistent with the conclusion made by Reid et al., as Cu^2+^ is strongly JT-active. However, Mn^2+^ (d^5^ electron configuration) and Zn^2+^ are not JT-active. Mn^2+^ is quite large (0.83 Å), and Fe^2+^ (0.78 Å) is the largest divalent cation successfully substituted in the Na*A*^2+^_0.5_Ti_1.5_O_4_ compositions. It is easy to conclude that the size mismatch between the divalent cation and Ti^4+^ becomes too large when *A*^2+^ = Mn^2+^, destabilizing the CF phase, but the results of the Na*A*^2+^_0.5_Sn_1.5_O_4_ system cast doubt on this explanation. On the other hand, Zn^2+^ has an ionic radius (0.74 Å) within the range of the divalent metals substituted into Na*A*^2+^_0.5_Ti_1.5_O_4_. It is possible that the preference of Zn^2+^ for tetrahedral sites destabilizes the CF structure. A compound with lower Na and Zn content in the Na–Zn–Ti–O system, freudenbergite-type Na_1.84_Zn_0.92_Ti_7.08_O_16_, does contain octahedrally coordinated Zn^2+^.^62^ However, the Zn^2+^ content is dilute compared to the Ti^4+^ in the freudenbergite compound. Furthermore, the increased sodium content in the CF compound would be expected to increase the covalency of the Zn–O bonds. More covalent Zn–O bonds are expected to favor tetrahedrally coordinated Zn^2+^.

No compounds of the type Na*A*^2+^_0.5_Sn_1.5_O_4_ have been reported previously. Given the existence of Na*A*^2+^_0.5_Ti_1.5_O_4_ compounds and Na-CFs containing Sn^4+^ (NaFeSnO_4_), it seemed probable Na*A*^2+^_0.5_Sn_1.5_O_4_ would also be stable. Notably, more CF-Na*A*^2+^_0.5_Sn_1.5_O_4_ compounds were found than CF-Na*A*^2+^_0.5_Ti_1.5_O_4_ compounds, with Mg^2+^, Mn^2+^, Co^2+^, Ni^2+^, Cu^2+^, Zn^2+^, and Cd^2+^ all forming Na-CF compounds when paired with Sn^4+^. As with NaMn^3+^SnO_4_, Sn^4+^ stabilizes the CF structure even when paired with the strongly JT-active Cu^2+^. Given the existence of NaMnSnO_4_ and NaCu_0.5_Sn_1.5_O_4_, it appears that spherical (JT-inactive) cations are not a necessary condition for a stable CF phase in the case of Na*A*^2+^_0.5_Sn_1.5_O_4_ and Na*A*^3+^SnO_4_. NaZn_0.5_Sn_1.5_O_4_ and NaMn_0.5_Sn_1.5_O_4_ can also be formed, unlike NaZn_0.5_Ti_1.5_O_4_ and NaMn_0.5_Ti_1.5_O_4_. It would be easy to conclude that NaMn_0.5_Sn_1.5_O_4_ forms because the difference in ionic radii between Sn^4+^ and Mn^2+^ is smaller than the difference between Ti^4+^ and Mn^2+^. However, we were also able to form NaCd_0.5_Sn_1.5_O_4_, and the difference in ionic radius between Cd^2+^ (0.95 Å) and Sn^4+^ (0.69 Å) is even larger than the difference between Mn^2+^ (0.83 Å) and Ti^4+^ (0.605 Å). The existence of NaCd_0.5_Sn_1.5_O_4_ makes Cd^2+^ the largest divalent framework cation known to occur in an Na-CF compound so far.

Clearly, Ti^4+^ and Sn^4+^ behave quite differently in the Na-CF phase space. Sn^4+^ stabilizes the CF structure more than Ti^4+^, as many of the cations that do not form a Na-CF with Ti^4+^ do form one when paired with Sn^4+^ instead, as is the case with NaMnSnO_4_, NaInSnO_4_, NaMn_0.5_Sn_1.5_O_4_, NaCu_0.5_Sn_1.5_O_4_, and NaZn_0.5_Sn_1.5_O_4_. The ability of Ti^4+^ to accommodate a high degree of octahedral distortion, owing to its unique combination of size and d^0^ electron configuration,^63−65^ creates a large number of competing phases in the Ti systems not present in the Sn systems. For example, the Na_2_O–MgO–TiO_2_ phase diagram contains at least eight reported quaternary phases (including NaMg_0.5_Ti_1.5_O_4_),^52,66^ whereas, to the best of our knowledge, the NaMg_0.5_Sn_1.5_O_4_ reported in this paper is the only known phase in the Na_2_O–MgO–SnO_2_ system. Since many of the phases containing highly distorted TiO_6_ octahedra do not have Sn^4+^ analogues, such as the freudenbergite structure,^67^ it becomes more likely that the CF phase is on the thermodynamic convex hull in the Na_2_O–*A*O/*A*_2_O_3_–SnO_2_ systems. Another obvious difference between Ti^4+^ and Sn^4+^ is their ionic radii (0.605 and 0.69 Å, respectively). This difference was invoked by Chiring et al. to explain the stability of NaMnSnO_4_.^25^ It was suggested Sn^4+^ exerted chemical pressure on Mn^3+^, stabilizing the octahedral configuration, as opposed to the square-pyramidal coordination observed in Na_4_Mn_4_Ti_5_O_18_. Alternatively, one could say the framework sites in NaMnSnO_4_ are larger than in the hypothetical NaMnTiO_4_, which enhances the stability of octahedral Mn^3+^. This reasoning might also be applied to NaZn_0.5_Sn_1.5_O_4_ to explain how pairing Zn^2+^ with Sn^4+^ achieves the desired octahedral coordination of Zn^2+^ instead of tetrahedral coordination. Notably, tetrahedral Zn^2+^ (0.60 Å) has a radius closer to that of octahedral Ti^4+^ (0.605 Å) than octahedral Sn^4+^ (0.69 Å), and octahedral Zn^2+^ (0.74 Å) has a radius closer to that of octahedral Sn^4+^ than octahedral Ti^4+^. Thus, mixing of Sn^4+^ with Zn^2+^ on octahedral sites seems more favorable, allowing for ZnO_6_ octahedra. However, it is difficult to say if the “stabilization” of the CF phase is not simply a result of the destabilization of competing phases such as Na_4_Mn_4_Ti_5_O_18_ upon Sn^4+^ substitution owing to the preference of Sn^4+^ for more symmetric octahedra or if both factors are important.

NaFe_1.5_Sb_0.5_O_4_ was the only reported Na-CF of the type Na*A*^3+^_1.5_*B*^5+^_0.5_O_4_. We found that CF phases also exist in the Na^+^-Sc^3+^-Sb^5+^-O^2–^ and Na^+^-In^3+^-Sb^5+^-O^2–^ systems. However, mixtures with the ideal compositions corresponding to NaSc_1.5_Sb_0.5_O_4_ and NaIn_1.5_Sb_0.5_O_4_ do not result in phase purity, at least under the synthetic conditions explored. In the case of Na_1.16_In_1.18_Sb_0.66_O_4_, some of the Na^+^ (1.02 Å) occupies the framework sites. It is likely the larger size of In^3+^ (0.800 Å) relative to Fe^3+^ (0.645 Å) allows the framework-site mixing. Sc^3+^ (0.745 Å) is somewhat smaller than In^3+^, which is consistent with “NaSc_1.5_Sb_0.5_O_4_” having a smaller degree of Na^+^ substitution on the octahedral sites. Interestingly, NaCr_1.5_Sb_0.5_O_4_ could not be prepared at either 900 or 950 °C. P3 phases in the Na_1–*x*_Cr_1–*x*/2_Sb_*x*/2_O_2_ (0.42 ≤ *x* ≤ 0.5) system exist which are very close in composition to the target NaCr_1.5_Sb_0.5_O_4_, so it is likely the enhanced stability of highly Na-vacant layered phases is responsible for the absence of NaCr_1.5_Sb_0.5_O_4_.^68^ In contrast, the layered Na_1–*x*_Fe_1–*x*/2_Sb_*x*/2_O_2_ phase is stable only down to *x* = 0.25, and the CF phase is observed when lower *x* values are attempted.^69^

While solid solutions between NaScTiO_4_ and NaFeTiO_4_ have been successfully synthesized, to the best of our knowledge, no other Na-CF solid solutions have been reported to form via ambient-pressure synthesis. We successfully synthesized three Na*A*^3+^*B*^4+^O_4_–Na*A*^2+^_0.5_*B*^4+^_1.5_O_4_ compositions: NaCo_1/3_Fe_1/3_Ti_4/3_O_4_, NaNi_1/3_Sc_1/3_Ti_4/3_O_4_, and NaNi_1/3_Fe_1/3_Ti_4/3_O_4_. While other solid solutions were not attempted, these results suggest numerous solid solution series are accessible.

Given the results presented here and in the context of previous literature, we suggest that the phase space of Na-CFs might be expanded even more through hydrothermal synthesis by the introduction of more cation site order and stoichiometry nonhomogeneity. Some of the Na-CFs reported here show cation preference between the two framework sites, even though the compounds were quenched from high temperature. If these can be synthesized via hydrothermal synthesis, it is likely the degree of cation preference and sodium vacancies can be enhanced, as in the case of some of the Na_0.55_Fe_0.28_Ti_1.72_O_4_ and Na_1–*x*_Fe_1–*x*_Ti_1+*x*_O_4_.^38,57^ More superstructures of the CF structure may also be discovered, such as in the case of Na_3_Mn_4_Te_2_O_12_,^39^ and would be more likely when the framework cations have greater differences in charge density. Finally, some cation combinations that do not produce a CF at high temperature might be stabilized under, e.g., hydrothermal conditions.

**Figure 7:**
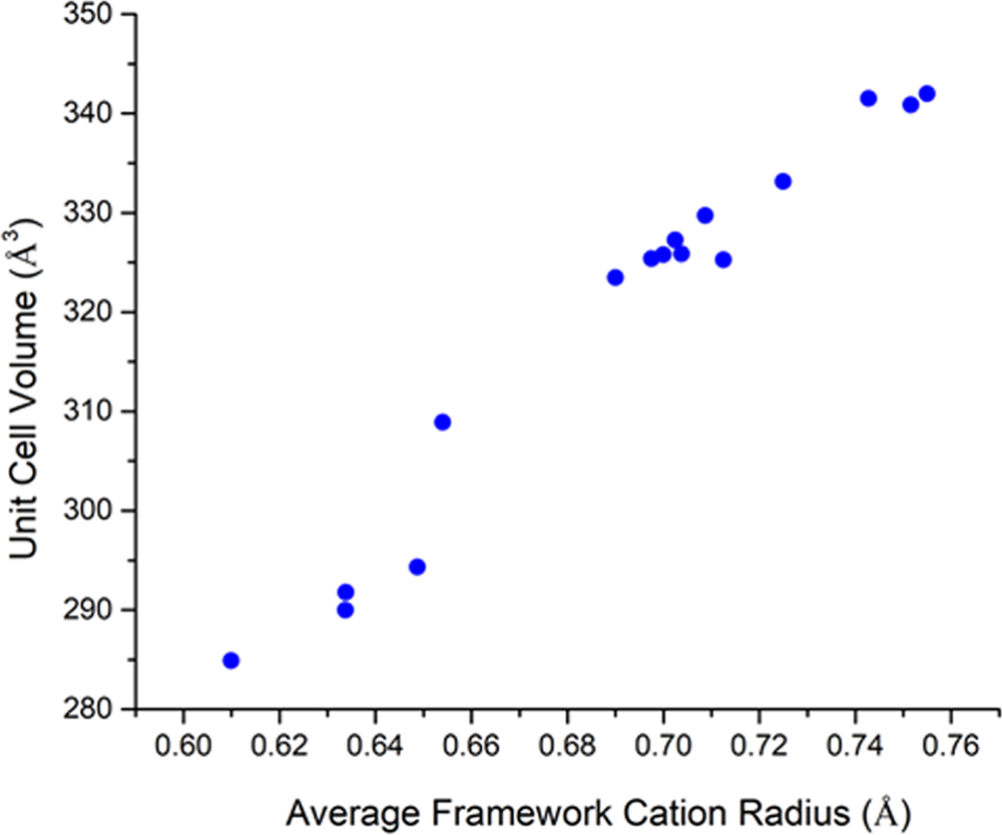
Unit cell volume of the new Na-CFs versus the weighted-average effective ionic radius of the framework cations in octahedral coordination.^58^

The unit cell volumes for the new compounds range from 284.872(1) Å^3^ for Na_0.99_Cr_0.99_Ti_1.01_O_4_ to 341.995(17) Å^3^ for NaCd_0.5_Sn_1.5_O_4_. As would be expected, the unit cell volume generally increases as the weighted-average framework cation radius increases (see Figure 7 and Table 1). The Na–O bond lengths for the NaO_8_ bicapped trigonal prisms increase as the unit cell volume increases. For Na_0.99_Cr_0.99_Ti_1.01_O_4_, the Na–O bond lengths range from 2.378(1) Å to 2.568(1) Å. For NaNi_0.5_Sn_1.5_O_4_, with a unit cell volume of 323.483(2) Å^3^, the Na–O bond lengths range from 2.444(2) Å to 2.644(2) Å. For Na_0.96_Sn_0.96_Sn_1.04_O_4_, with a unit cell volume of 341.504(2) Å^3^, the Na–O bond lengths range from 2.472(2) Å to 2.728(3) Å (see Table S2). This may have implications for Na^+^ mobility.

### Comparison to Lithium Spinels

A natural comparison to Na-CF compounds are the lithium spinels. Since Li^+^ is smaller than Na^+^, it favors lower coordination numbers and shorter bond distances to oxygen, stabilizing the spinel structure as opposed to the CF structure. The spinel structure contains two primary cation sites, the tetrahedral 8*a* site and the octahedral 16*d* site. The octahedral sites comprise the framework; a 3D series of interconnected tunnels are formed by the occupied tetrahedral sites and empty octahedral 16*c* sites. Because Li^+^ can readily accommodate coordination numbers of four and six, it can occupy both the tetrahedral 8*c* and octahedral 16*d* sites. This results in antisite defects, or inversion, in which Li^+^ and another cation that does not have a high octahedral site preference are statically distributed among the 8*c* and 16*d* sites according to their relative site preferences. In contrast, this does not occur in Na-CFs to a measurable extent. While Na^+^, like Li^+^, varies in its coordination number and geometry for known materials and is known to occupy both 6- and 8-coordinate sites; the smaller, more highly charged cations like Ti^4+^ are not expected to occupy 8-coordinate sites except at very high pressures. In addition, the larger difference in size between Na^+^ and the CF framework cations relative to Li^+^ and spinel framework cations increases the site preferences. Thus, in LiFeTiO_4_, significant inversion is observed, with Li^+^ and Fe^3+^ essentially being randomly distributed.^70^ In contrast, in NaFeTiO_4_, Na^+^ solely occupies the 8-coordinate tunnel sites, and Fe^3+^ and Ti^4+^ exclusively occupy the octahedral framework sites.^8^ For energy storage applications, the lack of antisite defects in Na-CF’s is expected to be beneficial.^71^ Inversion in spinels results in highly charged, immobile cations occupying the tetrahedral sites, which blocks the lowest energy diffusion path, hindering electrochemical performance.^72−74^ This likely explains why spinel-LiFeTiO_4_, with a high degree of inversion, has both a high activation energy for ionic conduction and a low reversible specific capacity,^70,75^ whereas CF-LiFeTiO_4_, synthesized by ion exchange from NaFeTiO_4_, nearly achieves theoretical capacity.^19^ Furthermore, Na^+^ is known to be exchangeable with Li^+^ topotactically for some Na-CFs, meaning Li-CFs can be accessed at ambient pressure.^19,20,23^

The ability of Li^+^ to occupy both 4- and 6- coordinate sites can also be exploited to synthesize Li-rich spinels such as Li_4_Mn_5_O_12_ and Li_4_Ti_5_O_12_, in which Li^+^ occupies all tetrahedral positions and some of the octahedral framework sites as well.^54,55^ Analogous Na-CFs, in which Na would occupy all the 8-coordinate tunnel sites and some of the octahedral sites, were previously unreported. Na_1.16_In_1.18_Sb_0.66_O_4_ appears to be the first such example, with ∼8% of the framework sites occupied by Na^+^, with Na–Sc–Sb–O and Na–Cd–Sn–O also appearing to be further phases with framework Na^+^. However, framework sodium appears to be nonexistent for Na-CFs with redox-active transition metal cations. This is likely because the redox-active transition metals found in CFs are significantly smaller than In^3+^/Cd^2+^/Sc^3+^, which decreases the average size of the framework sites.

#### Thermodynamic Calculations

**Figure 8:**
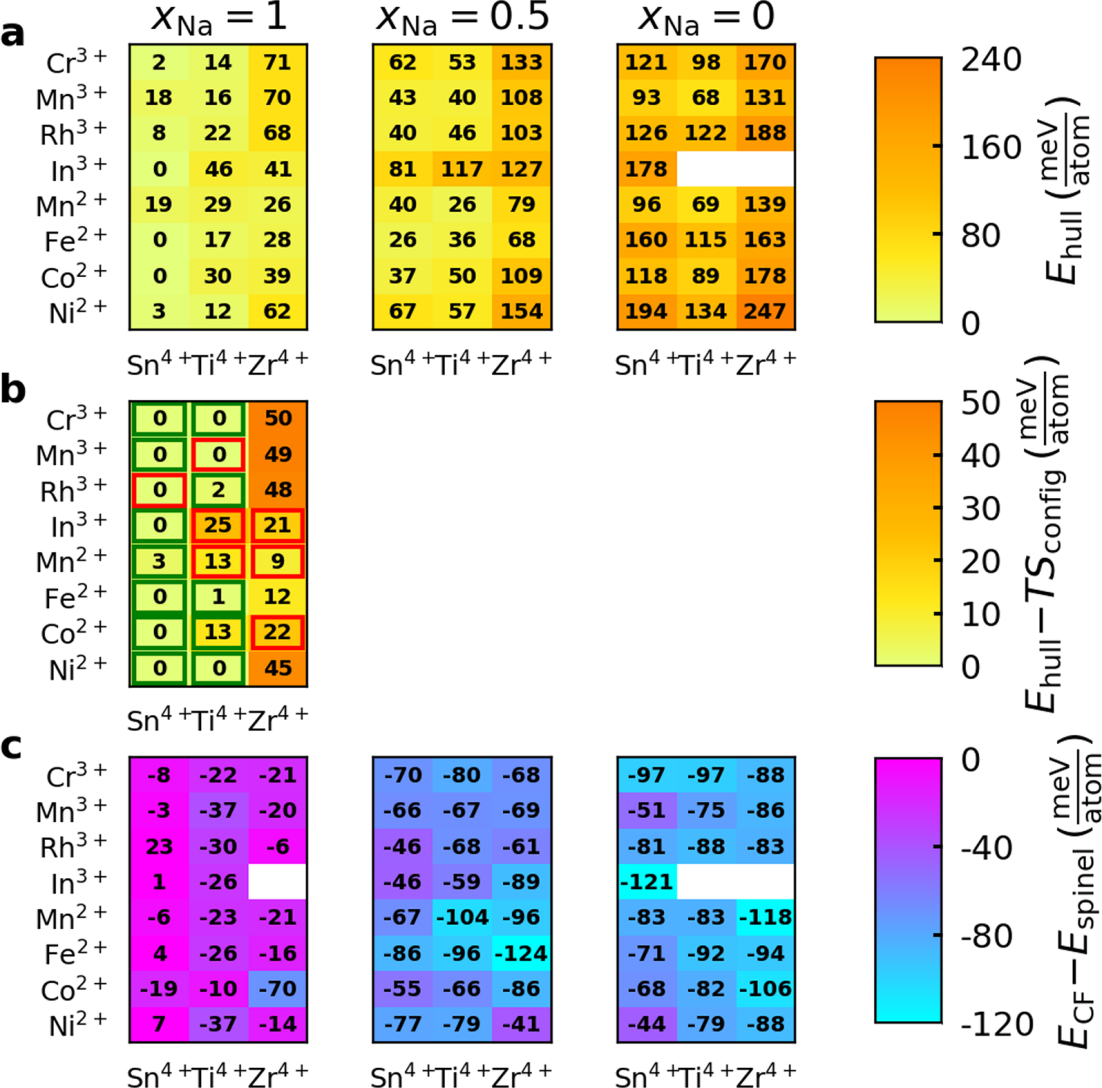
(a) Computed stability of Na_*x*_*A*^3+^*B*^4+^O_4_ and Na_*x*_*A*^2+^_0.5_*B*^4+^_1.5_O_4_ CF phases with three degrees of sodiation (*x* = 0, 0.5, 1.0). (b) Energy above hull of each *x*_Na_ = 1 compound after including ideal mixing entropy for the octahedral cations at 1200 K. Green and red boxes show successful/failed syntheses at ambient pressure. (c) Energy difference between the CF and spinel structure. Note that the empty boxes correspond to In-containing compounds that failed to converge in either the CF or spinel structures and were consequently excluded from this analysis.

Density functional theory (DFT) calculations were used to further understand the stability of phases in the Na-CF chemical space. The energy of the CF phase relative to the normal spinel phase (*Fd*3̅*m*) as well as the energy above the convex hull of stability, *E*_hull_, for the CF phases were computed for various combinations of octahedral cations and at varying levels of sodiation. Combinations were chosen to include a range of successfully synthesized Na-CFs as well as compositions not expected to produce a CF phase (e.g., NaCrZrO_4_). Calculated *E*_hull_ values of the Na-CF phase at 0 K and without considering entropic effects are shown in Figure 8a. If the two octahedral sites are equivalently occupied by the *A* and *B* cations in the Na-CF phases, ideal mixing would suggest configurational entropy provides significant stabilization at 1200 K (the approximate synthesis temperature), lowering the energy by ∼21 meV/atom for the Na*A*^3+^*B*^4+^O_4_ compositions and ∼17 meV/atom for the Na*A*^2+^_0.5_*B*^4+^_1.5_O_4_ compositions. Figure 8b shows *E*_hull_ after including this configurational entropy contribution for fully sodiated compounds. Each box in this panel is colored according to the experimentally observed synthesis ability, with green boxes indicating successful synthesis, red boxes indicating failed synthesis, and no box indicating no known synthesis attempt. When including configurational entropy, 8 of the 12 synthesized postspinels are predicted to be on the convex hull (stable), with three more lying very close to the hull (<5 meV/atom). The notable exception is NaCo_0.5_Ti_1.5_O_4_, which is calculated to lie 13 meV/atom above the hull. Of the six compounds that could not be synthesized in the postspinel structure, NaMnTiO_4_ and NaRhSnO_4_ are predicted to be stable, while the others are predicted to be unstable with respect to decomposition into competing phases. The thermodynamic stability of NaMnTiO_4_ and NaRhSnO_4_, which could not be synthesized, and the thermodynamic instability of NaCo_0.5_Ti_1.5_O_4_ emphasizes the role of kinetics and metastability in the synthesis of these oxides.^76,77^ We note that the calculated lack of stability of NaCo_0.5_Ti_1.5_O_4_ may arise from the inability to converge this structure with Co^2+^ in a high-spin state, as would be expected for CoO_6_ octahedra with Co^2+^ and was observed in our calculations for the other Co^2+^-containing CF phases (NaCo_0.5_Sn_1.5_O_4_ and NaCo_0.5_Zr_1.5_O_4_).

No new CF compounds containing Zr^4+^ were successfully synthesized, which is consistent with the high *E*_hull_ values calculated for the CF phases containing Zr^4+^ (ranging from 26 meV/atom for NaMn_0.5_Zr_1.5_O_4_ to 71 meV/atom for NaCrZrO_4_). After factoring in configurational entropy for the CF phase at 1200 K, all CF phases with Zr still remain above the hull. The stability of Na*A*^2+^_0.5_Zr_1.5_O_4_ increases as the size of *A*^2+^ increases, and NaMn_0.5_Zr_1.5_O_4_ is only 9 meV/atom above the hull when factoring in configurational entropy. The large size of Zr^4+^ and its ability to have coordination numbers higher than six likely destabilizes the CF structure relative to competing phases including ZrO_2_, in which Zr is 7-fold coordinated. In each attempted synthesis of the Na*A*^3+^ZrO_4_ and Na*A*^2+^_0.5_Zr_1.5_O_4_ phases, baddeleyite ZrO_2_ or a higher symmetry, partially substituted ZrO_2_ phase is formed as the main phase. NaScZrO_4_ appears to be a special case and remains the only known Na-CF containing Zr^4+^. Sn^4+^- and Ti^4+^-containing compounds have lower *E*_hull_ than those with Zr^4+^, in agreement with experiments that found many possible combinations including Sn and Ti. Considering that Sn^4+^ (0.69 Å) and Zr^4+^ (0.72 Å) have similar effective ionic radii, the increased stability of Sn^4+^-containing CF phases might originate because Sn^4+^ prefers the octahedral site more than Zr^4+^ or from the prevalence of low-energy zirconium oxide competing phases. Some of the newly synthesized phases contain redox-active metals and may be of interest as battery electrode materials, so thermodynamic calculations were also used to explore the (in)stability of the CF phase upon desodiation (Figure 8a). As expected, removing the Na^+^ cations destabilizes the CF structure to some degree, with values for the completely desodiated CF phases ranging from 68 meV/atom for MnTiO_4_ to 234 meV/atom for Ni_0.5_Zr_1.5_O_4_. The stability of the empty CF framework was compared to the empty spinel framework of the same composition (Figure 8c). Interestingly, the empty CF structure is more stable in every case examined here. Spinels are well-studied as Li-battery electrodes. While the spinel anodes like LiTi_2_O_4_, Li_4_Ti_5_O_12_, and LiCrTiO_4_ perform well when additional lithium is inserted and re-extracted,^78,79^ when full removal of the already-present Li^+^ is attempted, many lithium spinels either have limited capacity or show irreversible phase transitions that do not preserve the spinel lattice. This has been observed in LiTi_2_O_4_, LiV_2_O_4_, LiVTiO_4_, and LiCrTiO_4_.^79−82^ The calculations presented here indicate the CF framework is more stable when fully charged than the spinel framework. In fact, the charged CF-CrTiO_4_ phase has an *E*_hull_ about one-half that of the charged spinel-CrTiO_4_ phase (98 meV/atom vs 195 meV/atom). Given the variety of CF phases explored in these calculations, this is likely a general phenomenon and may extend to many more if not all CF/spinel compositions. Thus, CF phases appear to be promising as energy storage materials.

## Conclusions

The phase space of Na-containing CaFe_2_O_4_-type compounds has been expanded to include 16 new compositions and several additional solid-solutions. Previously it was suggested that only cations without Jahn–Teller distortions (spherical cations) form CaFe_2_O_4_-type compounds with sodium in the tunnels, but the existence of NaMnSnO_4_ and NaCu_0.5_Sn_1.5_O_4_ shows that this “requirement” is relaxed when *B*^4+^ is Sn^4+^. However, tetravalent cations larger than Sn^4+^ do not effectively stabilize the CaFe_2_O_4_ structure, and NaScZrO_4_ and NaScHfO_4_ remain the only known Na-CFs when the tetravalent cation is larger than Sn^4+^. In most cases, the framework cations are randomly distributed among the two framework sites, but weak site preference was observed for NaMg_0.5_Ti_1.5_O_4_ and NaCu_0.5_Sn_1.5_O_4_. Only in one case did Rietveld refinement and ^23^Na NMR spectroscopy indicate strong site preference: the first known CaFe_2_O_4_-type compound with Na^+^ occupying framework sites, Na_1.16_In_1.18_Sb_0.66_O_4_. In this material, the large Na^+^ cation strongly prefers one of the two symmetrically distinct framework sites, suggesting the order is driven by differences in charge density. “NaSc_1.5_Sb_0.5_O_4_,” which always contained secondary phases, may also contain Na^+^ in the framework sites, albeit to a lesser degree than Na_1.16_In_1.18_Sb_0.66_O_4_. DFT calculations revealed that most of the successfully synthesized Na-CFs were on or near their respective convex hull, with the exception of NaCo_0.5_Ti_1.5_O_4_, whereas the hypothetical Na-CFs containing Zr^4+^ were much higher in energy, suggesting the Na-CF compounds containing Zr^4+^ are thermodynamically unstable. Additional DFT calculations show that the charged (desodiated) CF framework is more stable than a charged spinel framework of the same composition, suggesting an opportunity for postspinel phases as Li/Na/Mg electrode materials. Given the picture described here, the richness of this phase space can likely be further expanded by synthesizing solid solutions as well as using hydrothermal and other soft chemical methods. The growing library of CaFe_2_O_4_-type materials inspires future fundamental and applied studies on these materials and related phase spaces.

## Supporting Information Available

Table of attempted syntheses and products, Rietveld refinements, additional ^23^Na NMR spectra, and table of Na–O bond distances for selected compounds (PDF)
