# Supplementary material for: Expanding the Ambient-Pressure Phase Space of CaFe2O4-Type Sodium Postspinel Host–Guest Compounds
Source: ACS Org Inorg Au. 2021 Sep 1;2(1):8–22. doi: 10.1021/acsorginorgau.1c00019 (PMC9954301; doi:10.1021/acsorginorgau.1c00019)
Supplement: Supplementary file 1 — gg1c00019_si_001.pdf [file gg1c00019_si_001.pdf]

Supporting Information

Expanding the ambient-pressure phase space of $CaFe_2O_4$-type sodium post-spinel host–guest compounds

Justin C. Hancock[1,2], Kent J. Griffith[1,2], Yunyeong Choi[2,3], Christopher J. Bartel[2,3], Saul H. Lapidus,[2,4] John T. Vaughey,[2,5] Gerbrand Ceder[2,3,6], Kenneth R. Poeppelmeier[1,2*]

[1]Department of Chemistry, Northwestern University, Evanston, Illinois 60208, United States
[2]Joint Center for Energy Storage Research, Argonne National Laboratory, Argonne, IL 60439, USA.
[3]Department of Materials Science and Engineering, University of California, Berkeley, California 94720, United States
[4]X-ray Science Division, Argonne National Laboratory, Argonne, IL 60439, USA
[5]Chemical Sciences and Engineering Division, Argonne National Laboratory, Lemont, IL, 60439, USA
[6]Materials Sciences Division, Lawrence Berkeley National Laboratory, Berkeley, CA, USA

Table S1 Compositions that did not form a CF structure

Target compound	Starting materials	Applied temperature (s) (°C)	Atmosphere	Major phases
$NaYZrO_4$	$NaHCO_3$, Y_2O_3, ZrO_2	1050<br><br>1200	Air	Na_2ZrO_3, Y_2O_3, cubic Y-substituted ZrO_2<br><br>cubic Y-substituted ZrO_2
$NaInZrO_4$	$NaHCO_3$, In_2O_3, ZrO_2	950<br><br>1100	Air	$NaInO_2$, ZrO_2<br><br>$NaInO_2$, In_2O_3, ZrO_2
$NaScRuO_4$	$NaHCO_3$, Sc_2O_3, RuO_2	950	Air	$Na_{2+x}Ru_4O_9$ (Sc-substituted?), Sc_2O_3
$NaMnRuO_4$	$NaHCO_3$, Mn_2O_3, RuO_2	950	Air	RuO_2, Na_xMnO_2
$NaRhRuO_4$	$NaHCO_3$, Rh_2O_3, RuO_2	950	Air	RuO_2, unknown- possibly $Na_x(Rh,Ru)O_2$
$NaAlSnO_4$	$NaHCO_3$, Al_2O_3, SnO_2	950	Air	SnO_2, $NaAlO_2$
$NaGaSnO_4$	$NaHCO_3$, Ga_2O_3, SnO_2	950<br><br>1200	Air	$NaGaO_2$, SnO_2<br><br>$NaGaO_2$, SnO_2
$NaYSnO_4$	$NaHCO_3$, Y_2O_3, SnO_2	1200	Air	$Y_2Sn_2O_7$, Y_2O_3
$NaRhSnO_4$	$NaRhO_2$, SnO_2	1000	Air	SnO_2, $NaRhO_2$
$NaMn_{0.5}Ti_{1.5}O_4$	$NaHCO_3$, MnO, TiO_2	875<br><br>950	Argon	$Na_2Ti_3O_7$, $MnTiO_3$, unknown<br><br>$Na_2Ti_3O_7$, $MnTiO_3$, unknown
$NaCu_{0.5}Ti_{1.5}O_4$	$NaHCO_3$, CuO, TiO_2	925	Air	$Na_2Ti_3O_7$, $Na_5Cu_{2.5}Ti_{6.5}O_{18}$, CuO
$NaZn_{0.5}Ti_{1.5}O_4$	$NaHCO_3$, ZnO, TiO_2	900	Air	$Na_2Ti_3O_7$, ZnO
$NaMn_{0.5}Zr_{1.5}O_4$	$NaHCO_3$, MnO, ZrO_2	1000	Argon	ZrO_2, MnO, Na_2ZrO_3
$NaCo_{0.5}Zr_{1.5}O_4$	$NaHCO_3$, Co_3O_4, ZrO_2	950	Air	ZrO_2, Na_2ZrO_3, CoO
$NaCd_{0.5}Zr_{1.5}O_4$	$NaHCO_3$, CdO, ZrO_2	1000	Air	Na_2ZrO_3, ZrO_2, CdO
$NaNi_{0.5}Ru_{1.5}O_4$	$NaHCO_3$, NiO, RuO_2	950	Air	RuO_2, unknown- possibly mixture of $Na_x(Ni,Ru)O_2$ phases
$NaCr_{1.5}Sb_{0.5}O_4$	$NaHCO_3$, Cr_2O_3, $NaSbO_3$	900<br><br>950	Argon	$Na_{0.58}Cr_{0.79}Sb_{0.21}O_2$, $NaSbO_3$<br><br>$Na_{0.58}Cr_{0.79}Sb_{0.21}O_2$, $NaSbO_3$
$NaMn_{1.5}Sb_{0.5}O_4$	$NaHCO_3$, Mn_2O_3, Sb_2O_3	1000	Air	$NaSbO_3$, unknown
$NaRh_{1.5}Sb_{0.5}O_4$	$NaHCO_3$, Rh_2O_3, Sb_2O_3	950	Air	$NaSbO_3$, unknown
$NaSc_{1.5}Ta_{0.5}O_4$	$NaHCO_3$, Sc_2O_3, Ta_2O_5	950	Air	$NaTaO_3$, Sc_2O_3
$NaCoSbO_4$	$NaHCO_3$, Co_3O_4, Sb_2O_3	1200	Air	$NaSbO_3$, $Co_{2.33}Sb_{0.67}O_4$

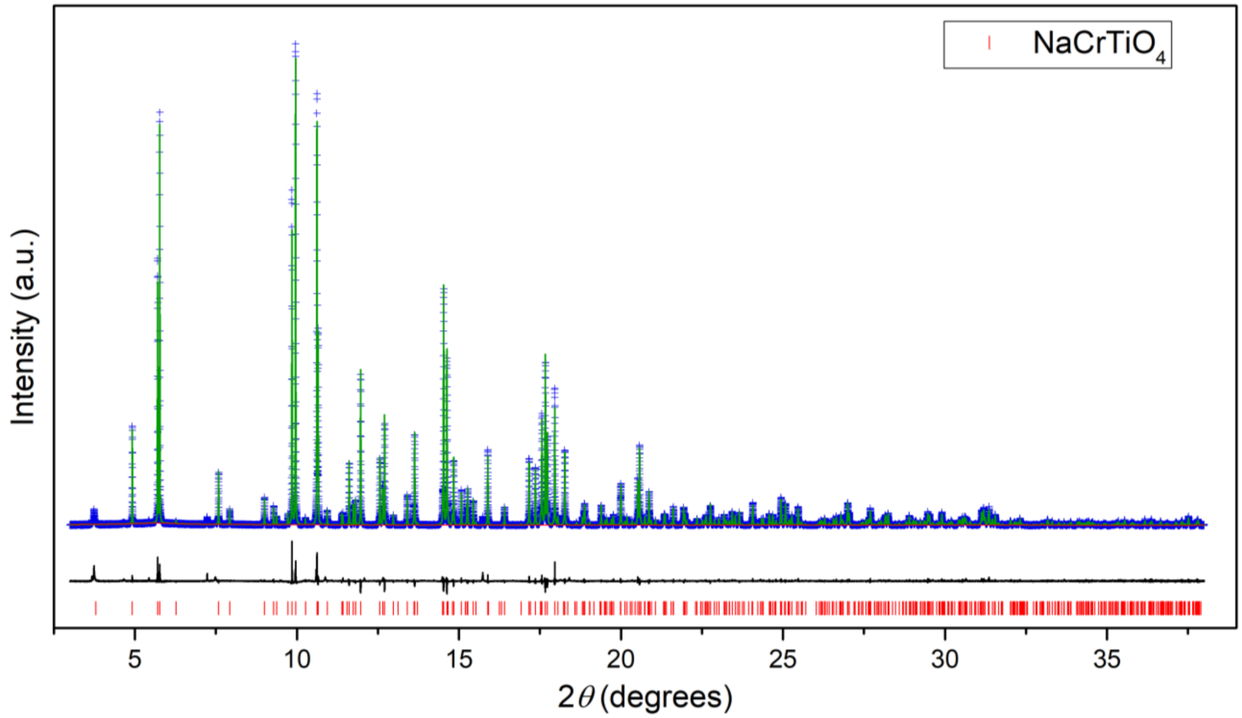

Figure S1 Rietveld refinement for $NaCrTiO_4$. Blue crosses are the observed intensities, the green curve is the fitted pattern, the black curve is the difference pattern, and the red tick marks indicate the location of the CF-$NaCrTiO_4$ peaks.

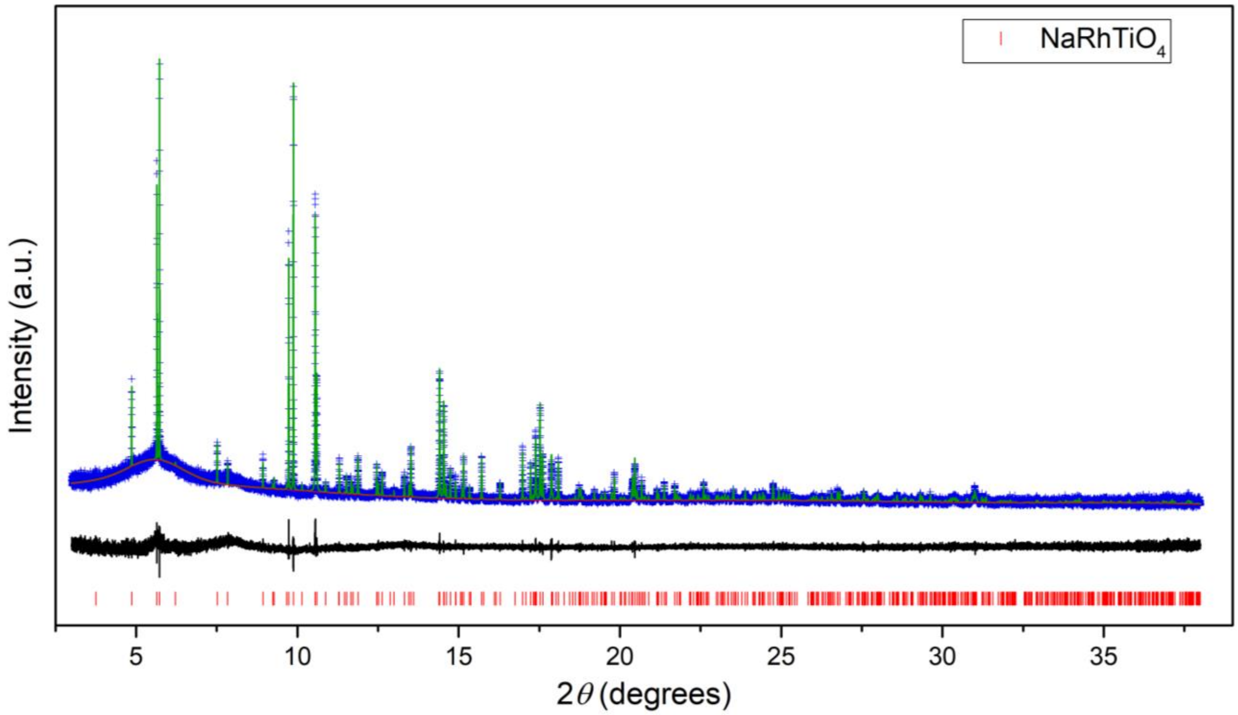

Figure S2 Rietveld refinement for $NaRhTiO_4$. Blue crosses are the observed intensities, the green curve is the fitted pattern, the black curve is the difference pattern, and the red tick marks indicate the location of the CF-$NaRhTiO_4$ peaks.

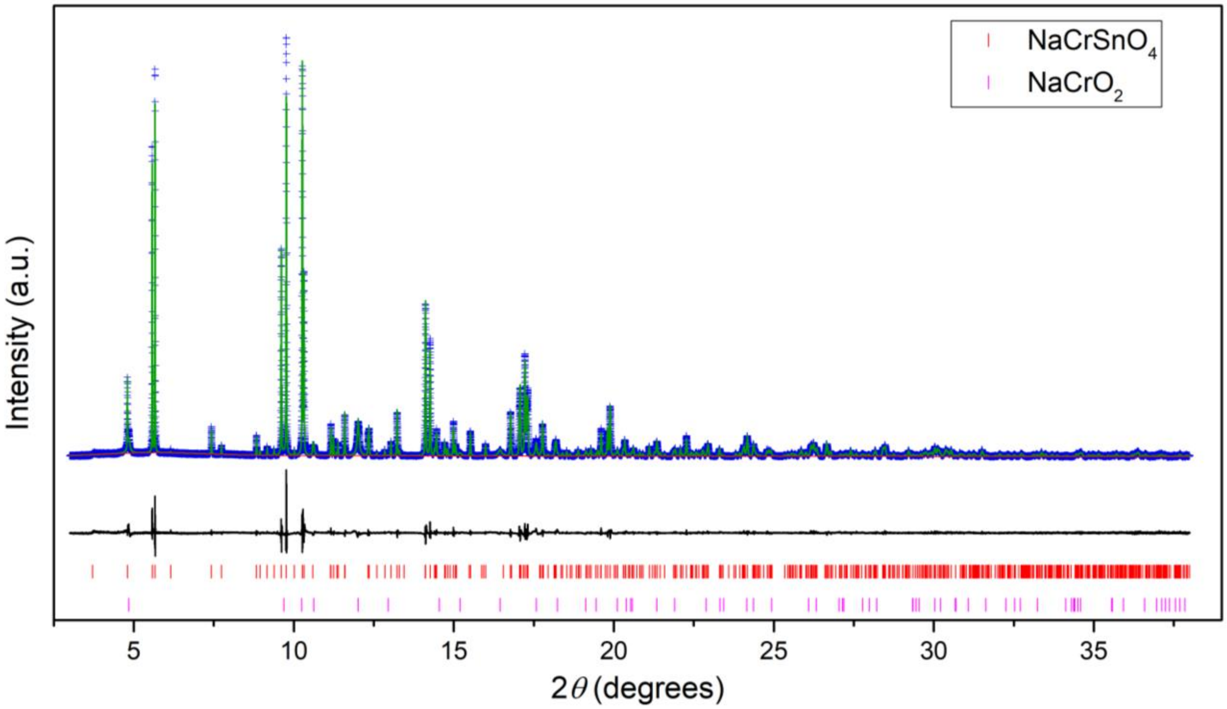

Figure S3 Rietveld refinement for $NaCrSnO_4$. Blue crosses are the observed intensities, the green curve is the fitted pattern, the black curve is the difference pattern, the red tick marks indicate the location of the CF-$NaCrSnO_4$ peaks, and the magenta tick marks indicate the location of $NaCrO_2$ peaks.

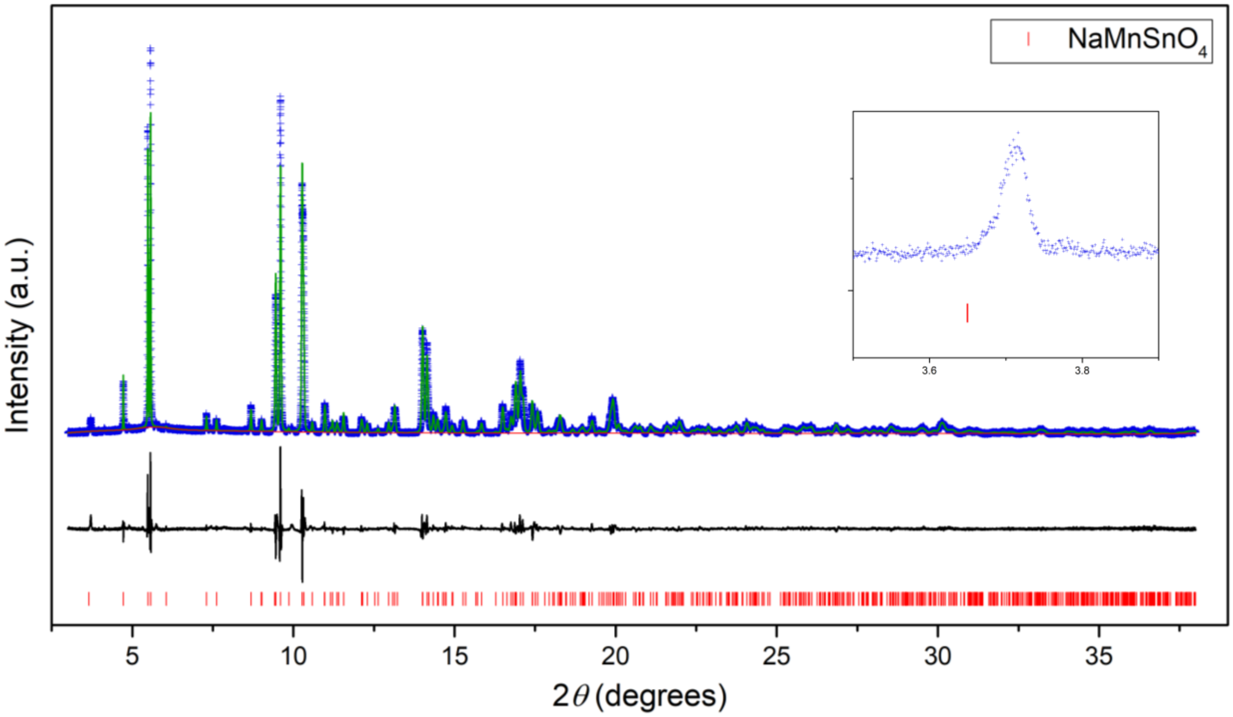

Figure S4 Rietveld refinement for $NaMnSnO_4$. Blue crosses are the observed intensities, the green curve is the fitted pattern, the black curve is the difference pattern, and the red tick marks indicate the location of the CF-$NaMnSnO_4$ peaks. The inset is included to show that the peak at 3.7° is an unknown impurity and too far away from the calculated angle of the (1 0 1) peak. Thus, cation site preference is either very weak or nonexistent in $NaMnSnO_4$.

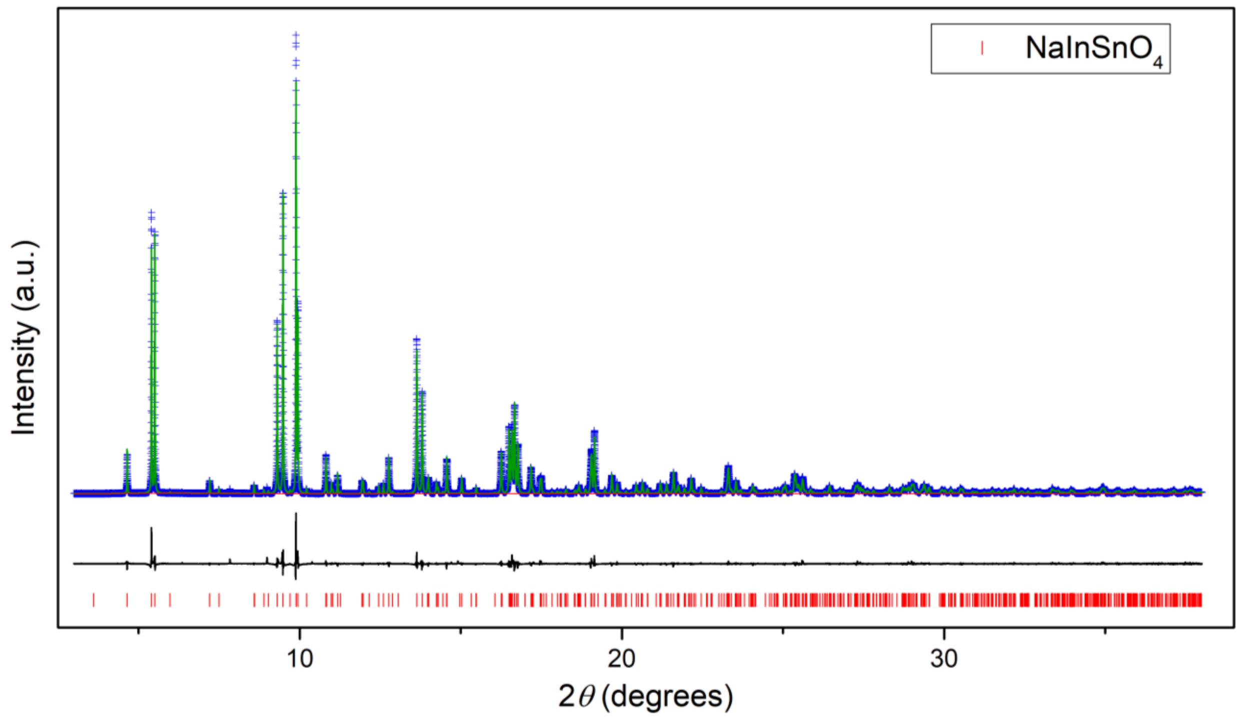

Figure S5 Rietveld refinement for $NaInSnO_4$. Blue crosses are the observed intensities, the green curve is the fitted pattern, the black curve is the difference pattern, and the red tick marks indicate the location of the CF-$NaInSnO_4$ peaks.

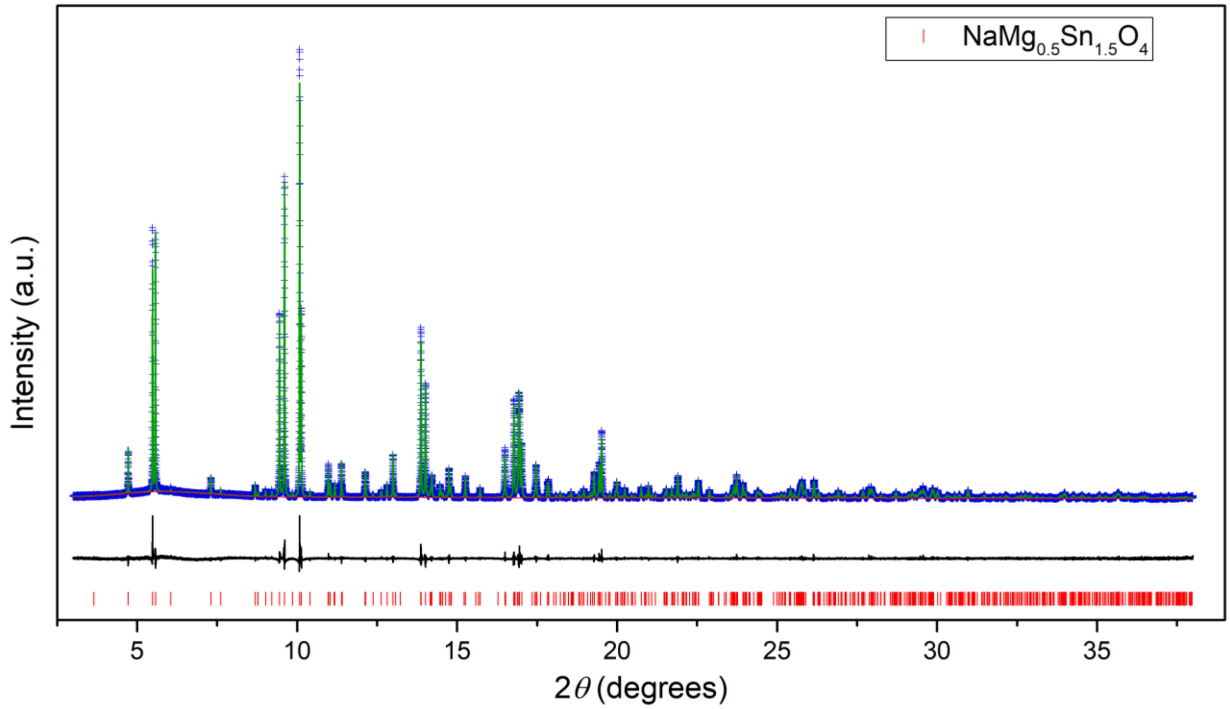

Figure S6 Rietveld refinement for $NaMg_{0.5}Sn_{1.5}O_4$. Blue crosses are the observed intensities, the green curve is the fitted pattern, the black curve is the difference pattern, and the red tick marks indicate the location of the CF-$NaMg_{0.5}Sn_{1.5}O_4$ peaks.

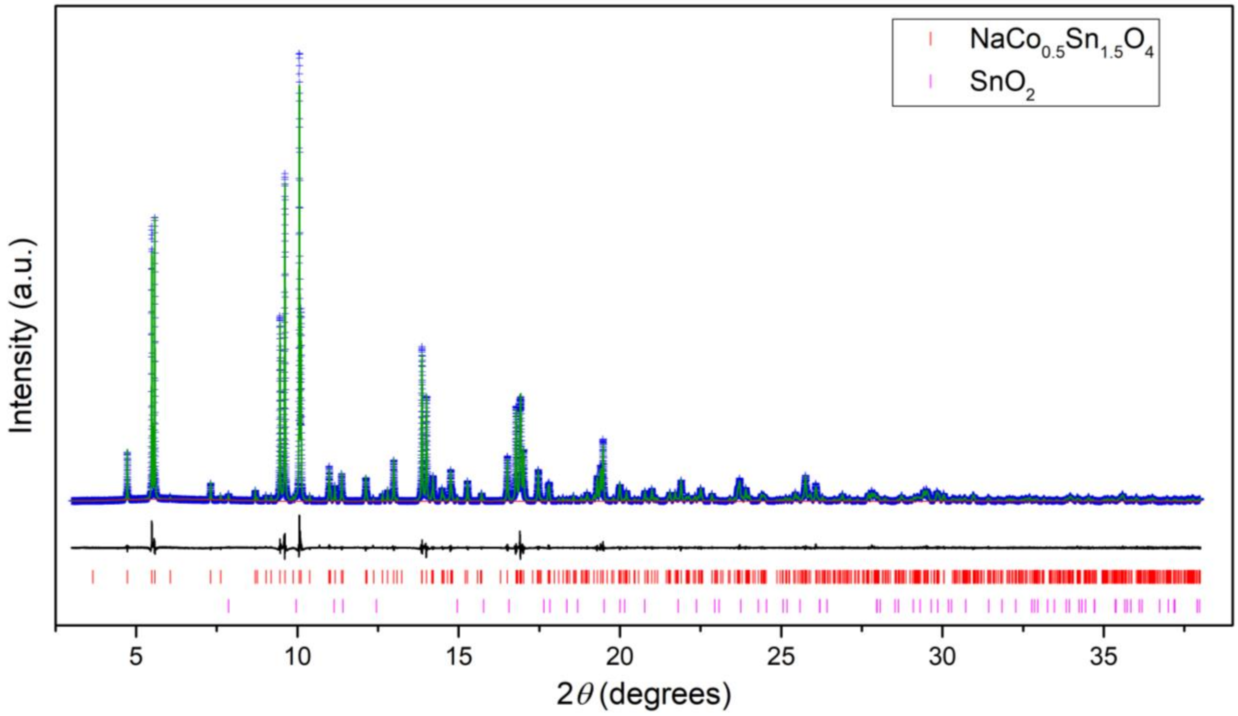

Figure S7 Rietveld refinement for $NaCo_{0.5}Sn_{1.5}O_4$. Blue crosses are the observed intensities, the green curve is the fitted pattern, the black curve is the difference pattern, the red tick marks indicate the location of the CF-$NaCo_{0.5}Sn_{1.5}O_4$ peaks, and the magenta tick marks indicate the location of SnO_2 peaks.

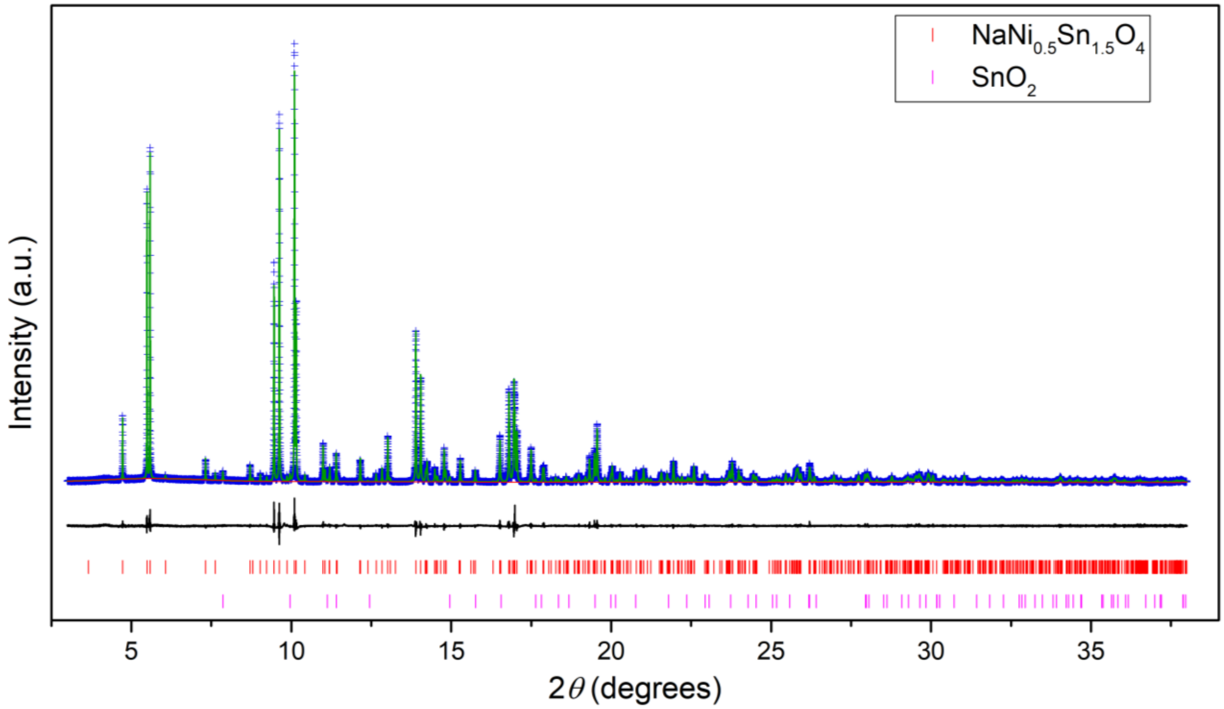

Figure S8 Rietveld refinement for $NaNi_{0.5}Sn_{1.5}O_4$. Blue crosses are the observed intensities, the green curve is the fitted pattern, the black curve is the difference pattern, the red tick marks indicate the location of the CF-$NaNi_{0.5}Sn_{1.5}O_4$ peaks, and the magenta tick marks indicate the location of SnO_2 peaks.

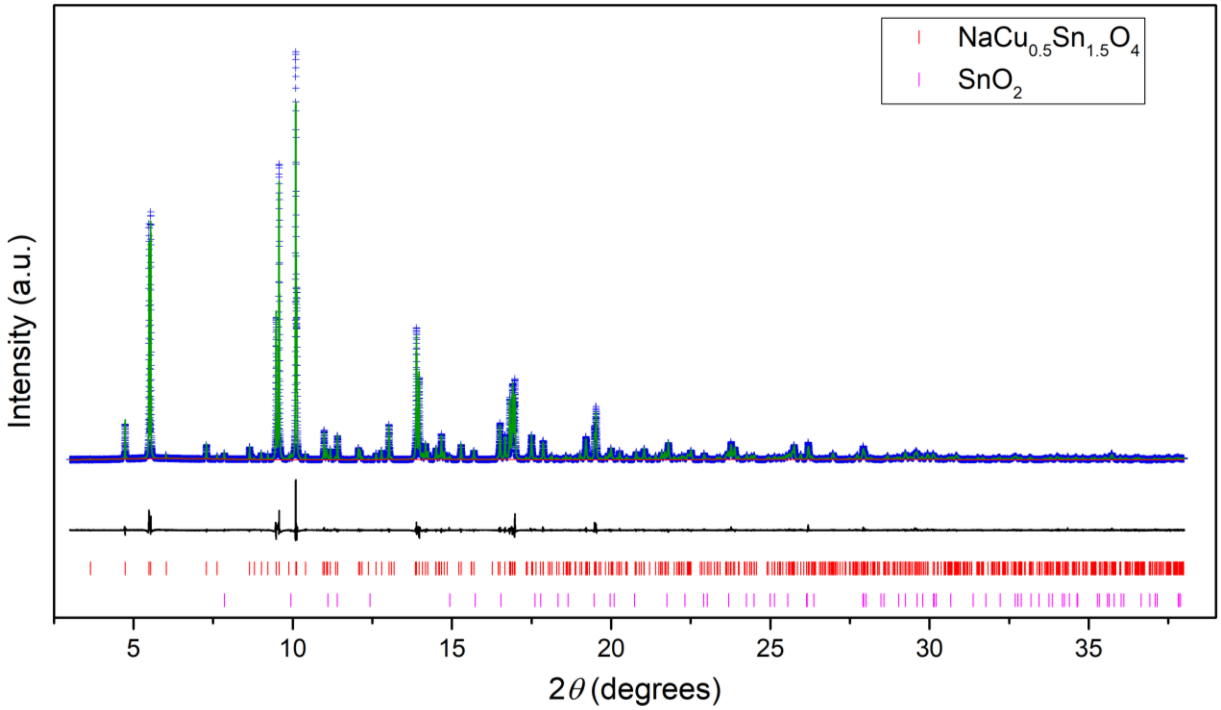

Figure S9 Rietveld refinement for $NaCu_{0.5}Sn_{1.5}O_4$. Blue crosses are the observed intensities, the green curve is the fitted pattern, the black curve is the difference pattern, the red tick marks indicate the location of the CF-$NaCu_{0.5}Sn_{1.5}O_4$ peaks, and the magenta tick marks indicate the location of SnO_2 peaks.

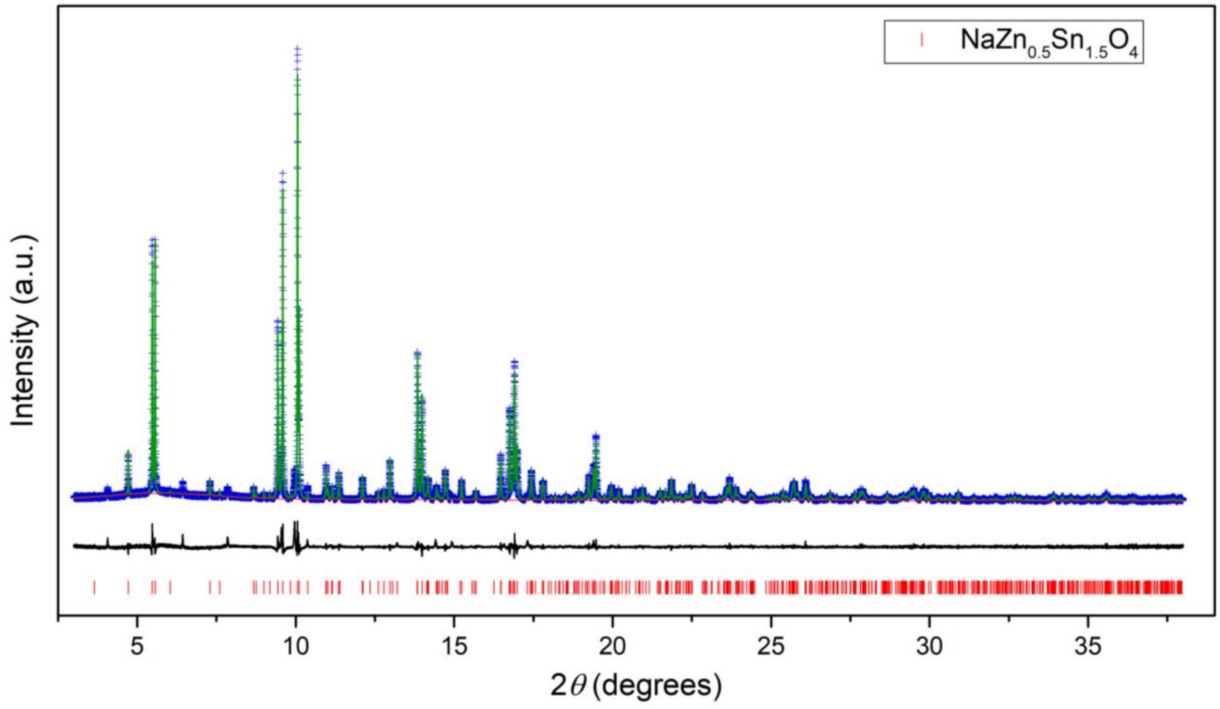

Figure S10 Rietveld refinement for $NaZn_{0.5}Sn_{1.5}O_4$. Blue crosses are the observed intensities, the green curve is the fitted pattern, the black curve is the difference pattern, and the red tick marks indicate the location of the CF-$NaZn_{0.5}Sn_{1.5}O_4$ peaks.

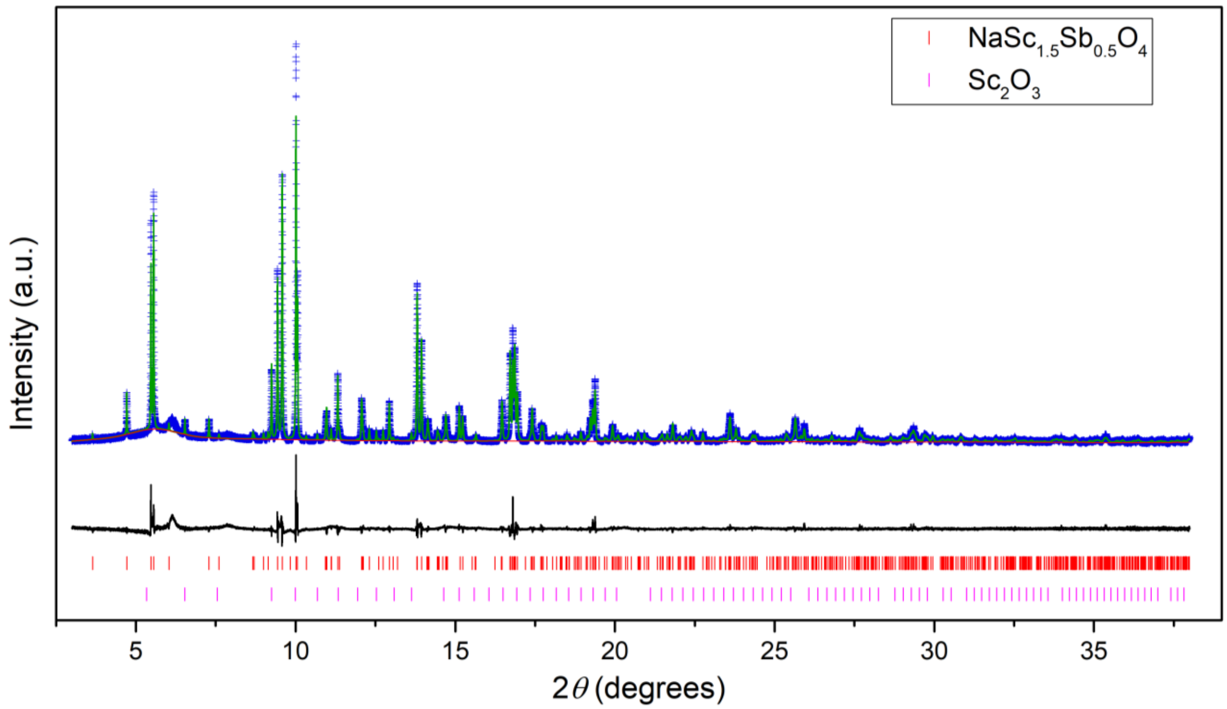

Figure S11 Rietveld refinement for “$NaSc_{1.5}Sb_{0.5}O_4$.” Blue crosses are the observed intensities, the green curve is the fitted pattern, the black curve is the difference pattern, the red tick marks indicate the location of the CF- $NaSc_{1.5}Sb_{0.5}O_4$ peaks, and the magenta tick marks indicate the location of Sc_2O_3 peaks.

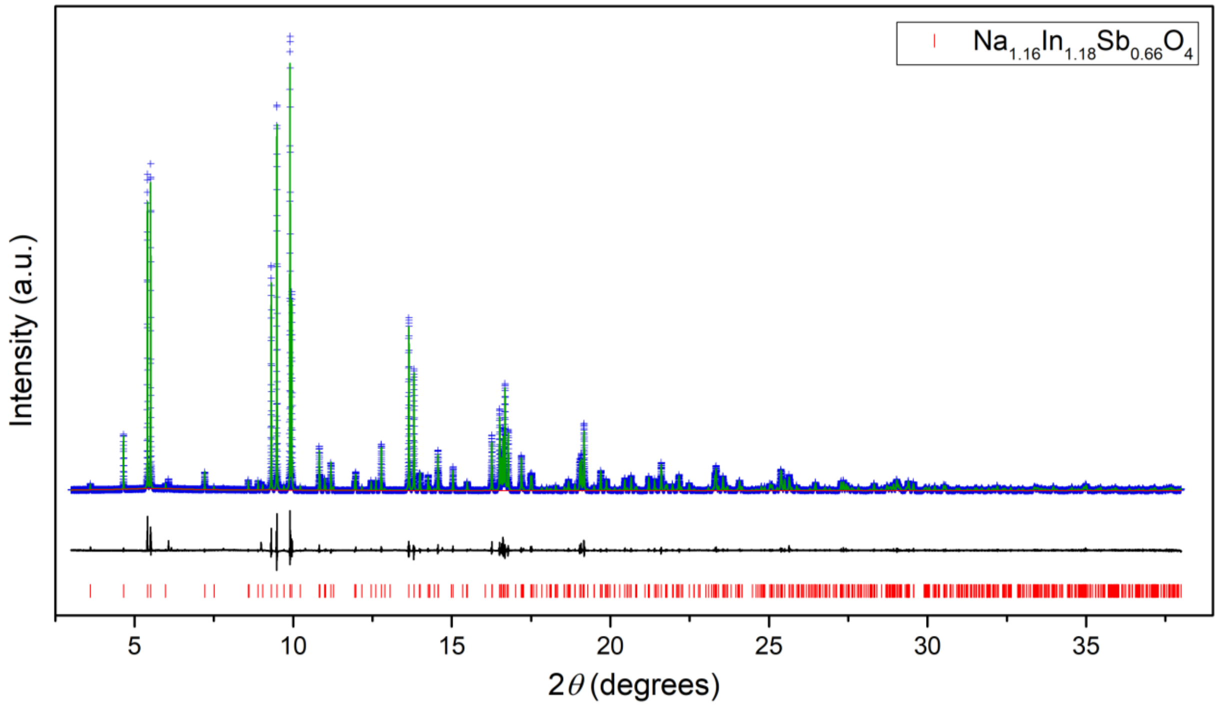

Figure S12 Rietveld refinement for $Na_{1.16}In_{1.18}Sb_{0.66}O_4$. Blue crosses are the observed intensities, the green curve is the fitted pattern, the black curve is the difference pattern, and the red tick marks indicate the location of the CF- $Na_{1.16}In_{1.18}Sb_{0.66}O_4$ peaks.

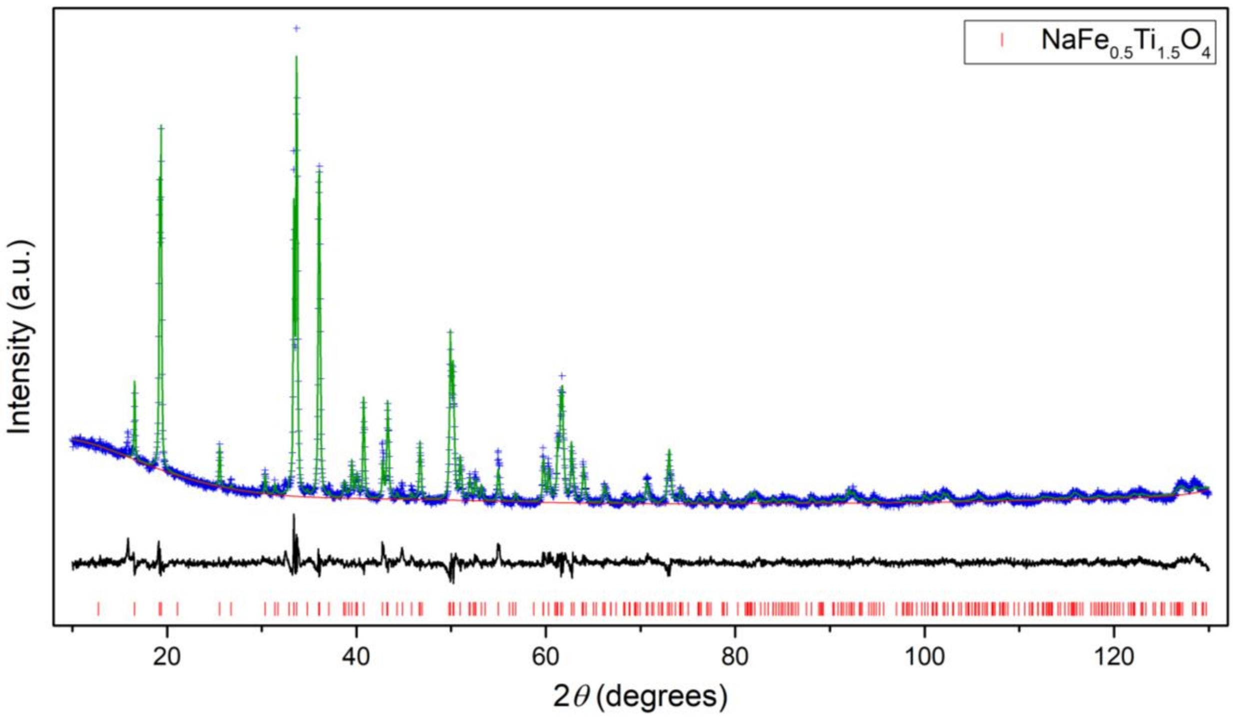

Figure S13 Rietveld refinement for $NaFe_{0.5}Ti_{1.5}O_4$. Blue crosses are the observed intensities, the green curve is the fitted pattern, the black curve is the difference pattern, and the red tick marks indicate the location of the CF-$NaFe_{0.5}Ti_{1.5}O_4$ peaks.

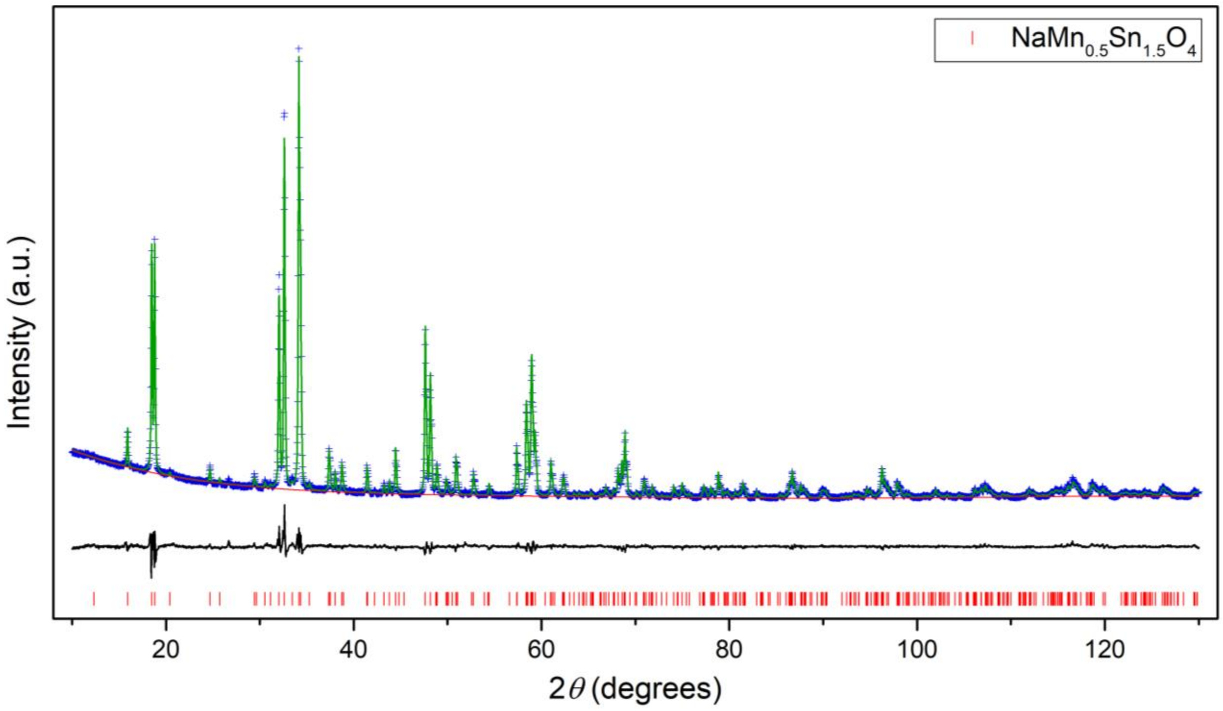

Figure S14 Rietveld refinement for $NaMn_{0.5}Sn_{1.5}O_4$. Blue crosses are the observed intensities, the green curve is the fitted pattern, the black curve is the difference pattern, and the red tick marks indicate the location of the CF-$NaMn_{0.5}Sn_{1.5}O_4$ peaks.

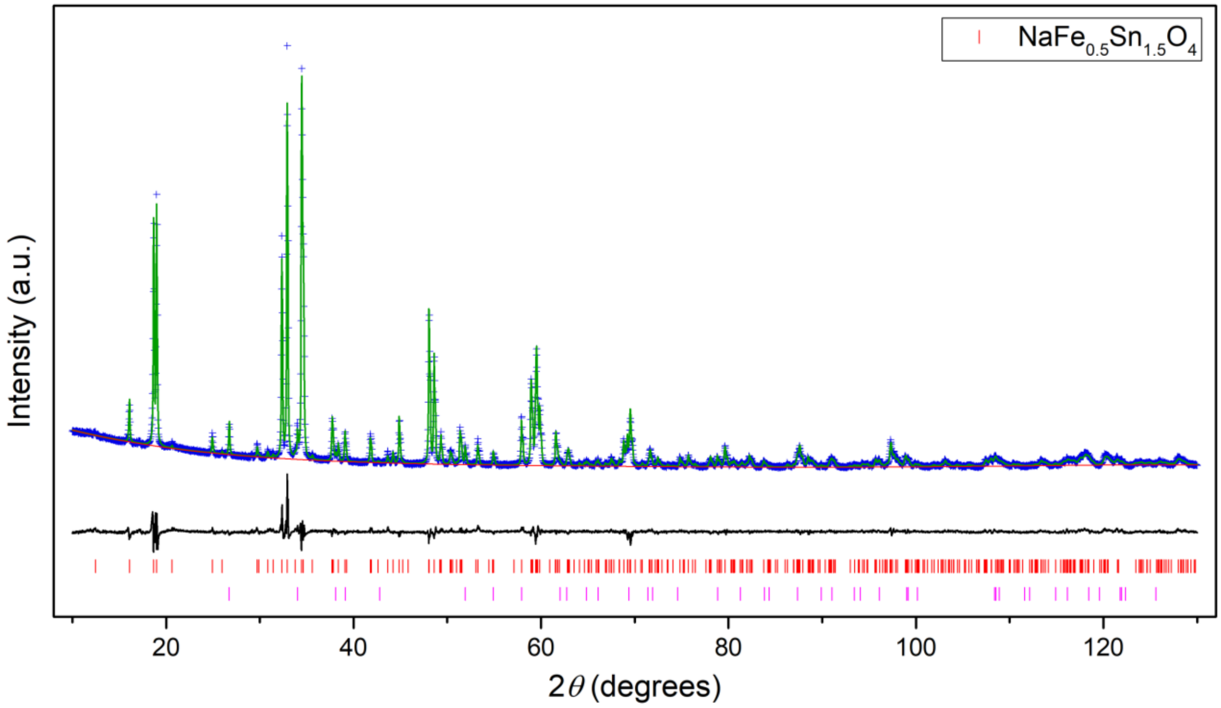

Figure S15 Rietveld refinement for $NaFe_{0.5}Sn_{1.5}O_4$. Blue crosses are the observed intensities, the green curve is the fitted pattern, the black curve is the difference pattern, the red tick marks indicate the location of the CF-$NaFe_{0.5}Sn_{1.5}O_4$ peaks, and the magenta tick marks indicate the location of SnO_2 peaks.

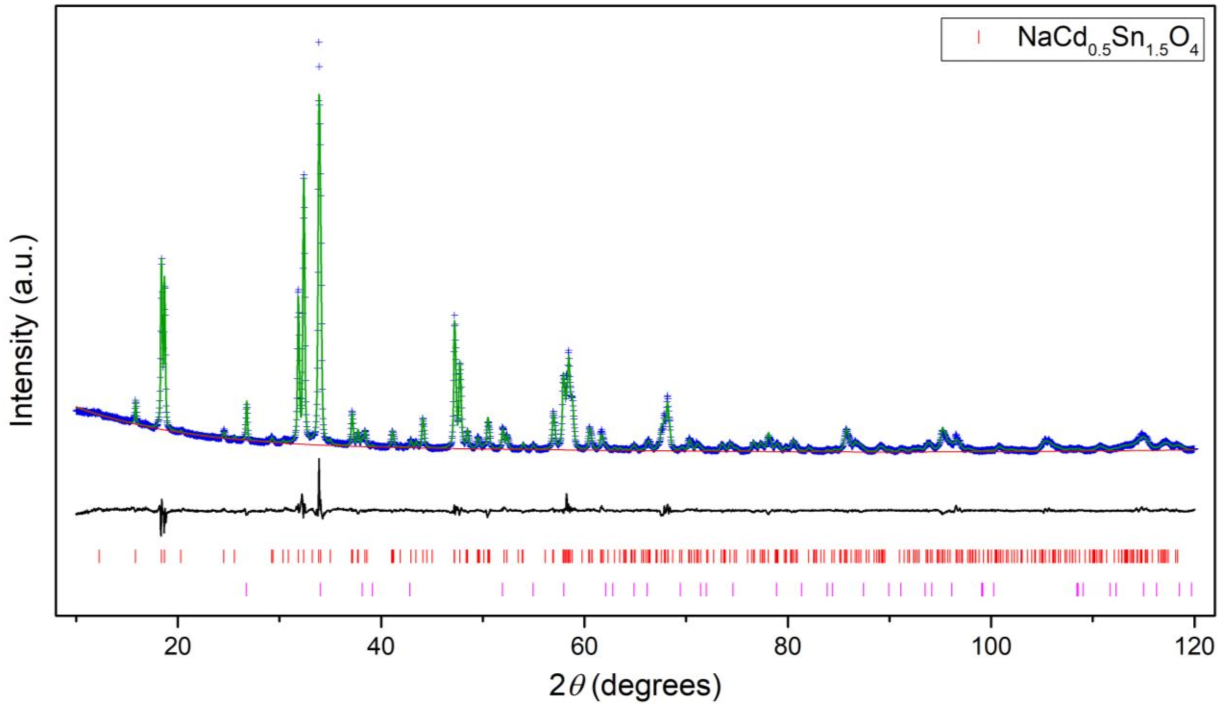

Figure S16 Rietveld refinement for $NaCd_{0.5}Sn_{1.5}O_4$. Blue crosses are the observed intensities, the green curve is the fitted pattern, the black curve is the difference pattern, the red tick marks indicate the location of the CF-$NaCd_{0.5}Sn_{1.5}O_4$ peaks, and the magenta tick marks indicate the location of SnO_2 peaks.

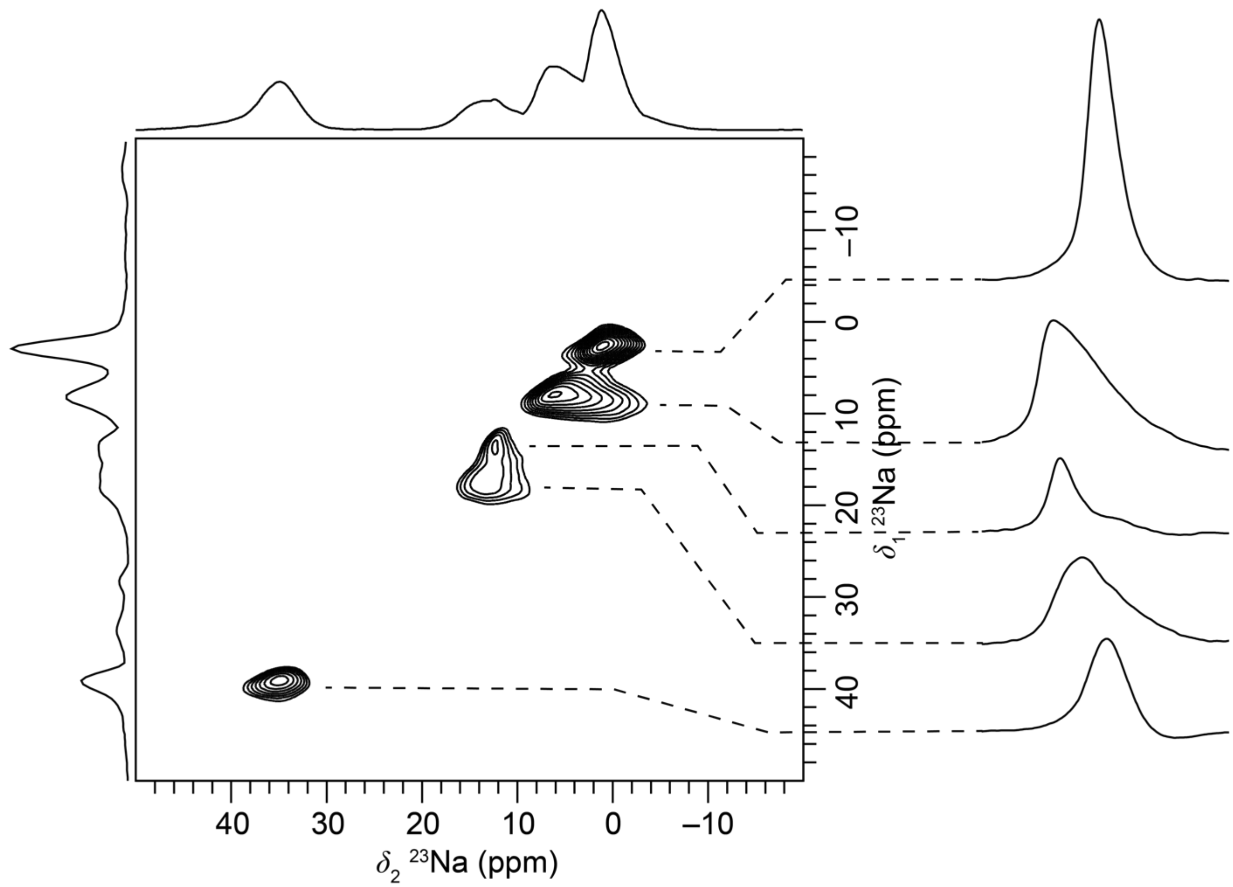

Figure S17 ^{23}Na Multiple-quantum MAS NMR spectrum of $Na_{1.16}In_{1.18}Sb_{0.66}O_4$ recorded with a z-filtered pulse sequence at 14 kHz MAS and 9.4 T. Cross sections, extracted parallel to δ_2, are shown on the right. Distinct quadrupolar lineshape features are not visible for any of the sodium environments.

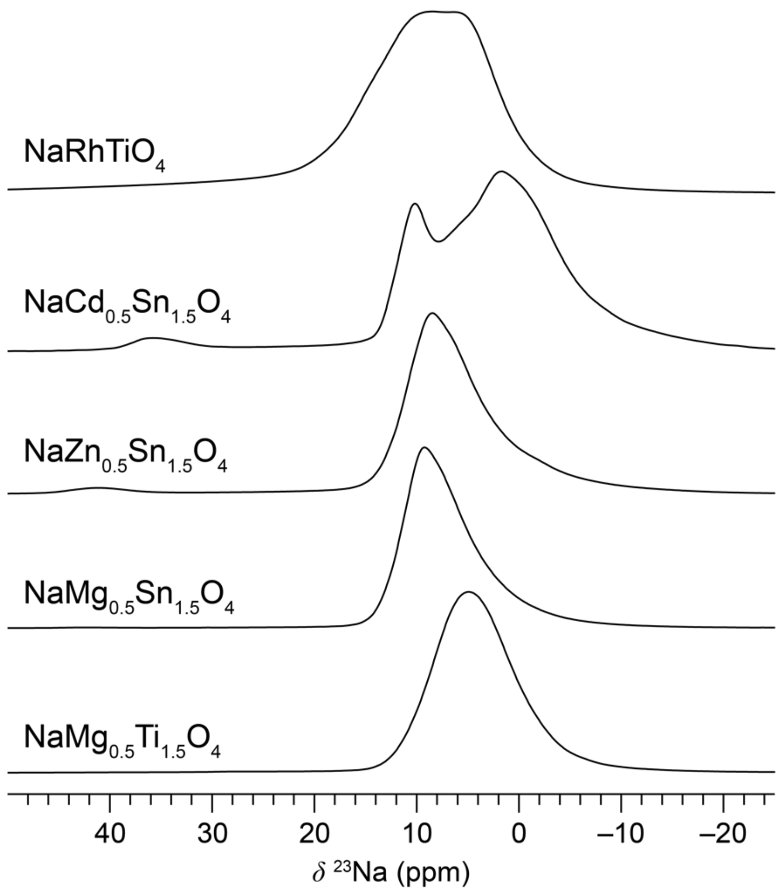

Figure S18 *^{23}Na Solid-state NMR of diamagnetic CF structures at 12.5 kHz MAS and 9.4 T.*

Table S2 Table of Na-O bond lengths for selected compounds

	$Na_{0.99}Cr_{0.99}Ti_{1.01}O_4$	$NaNi_{0.5}Sn_{1.5}O_4$	$Na_{0.96}In_{0.96}Sn_{1.04}O_4$
Na1-O2 (×2)	2.378(1) Å	2.444(2) Å	2.472(2) Å
Na1-O4 (×2)	2.390(1) Å	2.458(2) Å	2.484(2) Å
Na1-O3	2.507(1) Å	2.606(3) Å	2.674(3) Å
Na1-O3	2.533(1) Å	2.642(3) Å	2.701(3) Å
Na1-O1 (×2)	2.568(1) Å	2.644(2) Å	2.728(3) Å
